# Identification and Sensory Characterization of Umami Peptides During Lager Beer Fermentation

**DOI:** 10.3390/foods15101694

**Published:** 2026-05-12

**Authors:** Yashuai Wu, Wenjing Tian, Wanqiu Zhao, Jiayang Luo, Xin Yuan, Jiang Xie, Bofeng Zhong, Dongrui Zhao

**Affiliations:** 1 Department of Food and Bioengineering, Beijing Vocational College of Agriculture, Beijing 102442, China; wyss995418706@163.com (Y.W.); 18401344071@163.com (W.Z.); b230690090@163.com (J.L.); 2 School of Food Science and Engineering, South China University of Technology, Guangzhou 510640, China; 3 Key Laboratory of Geriatric Nutrition and Health, Beijing Technology and Business University, Ministry of Education, Beijing 100048, China; yuanxin5215@163.com (X.Y.); xj2445580935@sina.com (J.X.); z125678hhh@outlook.com (B.Z.); zdr@btbu.edu.cn (D.Z.); 4 Key Laboratory of Brewing Molecular Engineering of China Light Industry, Beijing Technology and Business University, Beijing 100048, China; 5 National Baijiu and Huangjiu Research Institute, Beijing Technology and Business University, Beijing 100048, China; 6 China Food Flavor and Nutrition Health Innovation Center, Beijing Technology and Business University, Beijing 100048, China

**Keywords:** lager beer, umami peptides, molecular docking, molecular simulation, molecular sensomics, full fermentation process

## Abstract

Despite increasing interest in beer-derived umami peptides, their formation during lager beer fermentation and their potential roles in receptor recognition and sensory perception remain insufficiently understood. Therefore, this study combined peptidomics, in silico screening, receptor-oriented analysis, and sensory evaluation to investigate the dynamic changes in umami-related peptides throughout lager beer fermentation and to explore their possible sensory relevance. Peptide profiles varied markedly across the fermentation process, indicating continuous proteolysis and dynamic restructuring of the peptide pool. From these profiles, candidate umami peptides were screened, and representative short peptides with plausible receptor-binding characteristics were selected for further evaluation. These peptides showed measurable taste activity and were mainly associated with modulation of the umami–bitterness–aftertaste dimension, whereas their effects on aroma and overall balance were limited. In addition, the abundance of umami-related peptides was positively associated with perceived umami intensity. Overall, the results suggest that fermentation-derived peptides may contribute to taste perception in lager beer and provide a basis for further studies on flavor-oriented brewing and process regulation.

## 1. Introduction

Lager beer is widely appreciated for its relatively clean and balanced flavor profile [1,2,3]. However, beyond volatile aroma compounds, nitrogenous components generated during brewing and fermentation, particularly peptides, are increasingly recognized as important contributors to taste expression and flavor quality. Proteolysis during the brewing process continuously reshapes the beer peptide pool, yet the dynamic formation of umami peptides in lager beer and their relationships with receptor recognition and sensory contribution remain insufficiently understood.

With increasing consumer demand for quality, the beer market has shifted from volume growth to quality-oriented competition, with greater emphasis on flavor consistency, mouthfeel, and nutritional value [4,5]. In this context, improving the sensory quality of lager beer has become an important industry objective [6,7]. Among taste attributes, umami is increasingly recognized as a relevant contributor to roundness, fullness, and flavor persistence in beer, mainly through fermentation-derived free amino acids and nucleotides [2,8,9,10,11,12].

However, in-depth understanding of the mechanisms underlying the umami characteristics of beer remains limited, and the molecular basis had not been precisely elucidated. Although recent studies have begun to identify umami peptides in finished lager beer and to explore their interactions with the T1R1/T1R3 receptor, these investigations have mainly focused on peptide discovery at the end-product level rather than on their dynamic formation and evolution throughout fermentation [5,13,14,15]. Analyses of the lager brewing process have mainly focused on volatile backbone substances (higher alcohols, esters) and odor-active compounds (OAV > 1), while tracking of taste substances—especially umami peptides—across fermentation is uncommon. In particular, a systematic process-wide understanding linking malt proteolysis, yeast autolysis, and lagering/maturation with peptide generation, receptor recognition, and sensory contribution is still lacking [5]. This unresolved connection constitutes the key research gap addressed in the present study. Existing evidence suggests that brewing raw materials such as malt and rice can generate short peptides, including acidic sequences potentially related to umami perception; however, their temporal evolution and mechanistic contribution to beer umami expression remain insufficiently clarified. Clarifying these relationships between process, peptide evolution, receptor interaction, and sensory output held significant theoretical and industrial importance [16].

Regrettably, the complex matrix of lager beer posed major challenges to the extraction, isolation, and identification of umami peptides, and traditional methods such as gel filtration chromatography (GFC) were time-consuming, costly, and low-throughput [17,18,19]. To improve screening efficiency, recent studies have introduced machine learning tools for candidate prescreening [20], while molecular docking has provided a complementary approach for evaluating peptide–receptor recognition before experimental validation [21]. Combined with sensomics-oriented analysis, these strategies help to relate predicted candidates to their potential sensory relevance in complex food systems [20,21].

Accordingly, the objective of this study was to characterize the dynamic formation of umami peptides throughout the entire fermentation process of lager beer and to elucidate their relationships with receptor recognition and sensory expression. Specifically, this study aimed to track temporal changes in umami-peptide profiles during fermentation, identify representative peptides with potential binding affinity for the T1R1/T1R3 umami receptor, and clarify the associations between peptide abundance and multidimensional sensory attributes. To achieve these aims, peptide profiling, bioinformatic prediction, molecular docking, and sensory evaluation were integrated to construct a quantitative framework linking peptide generation, receptor interaction, and sensory perception. Therefore, the main objective of this study was to elucidate the structure–activity evolution of umami peptides across the full fermentation process of lager beer, providing a molecular basis for flavor-oriented process control.

## 2. Materials and Methods

### 2.1. Samples and Reagents

The experimental samples were beer produced from 8 °P wort (ingredients: water, malt, rice, and hops) and supplied by Beijing Yanjing Beer Co., Ltd. (Beijing, China). Routine production was carried out in three independent brewing batches (n = 3), rather than as analytical replicates from a single batch, and all six fermentation timepoints were sampled from each independent fermentation run. The wort was cooled to 18.0 ± 0.5 °C before transfer to the fermentation tank and was continuously oxygenated during cooling at approximately 1 L/min. Fermentation was carried out using Angel Yeast BF16 lager yeast, a manufacturer-designated lager brewing strain for lager and Pilsen-style beers. According to the manufacturer, BF16 is classified as *Saccharomyces pastorianus* and is characterized by a fermentation temperature range of 10–20 °C, apparent attenuation of 80–84%, a POF-negative phenotype, negative diastatic activity, and high flocculation. Samples were collected on fermentation day 0 (wort), 1, 3, 10, 17, and 29, thereby covering the main stages from wort to fermentation and maturation. Primary fermentation was conducted at 18.0 ± 0.5 °C and 0.03 MPa for 2–3 days until tank sealing. During this period, the monitored extract decreased from the initial 8 °P to 4.3 ± 0.2 °Bx, at which point the tank was sealed and maintained at 18 °C and 0.12 MPa for 2–3 days for reduction. This was followed by maturation at 0 °C and 0.13 ± 0.01 MPa for 1–3 days. For the present study, analytical samples were withdrawn directly from the fermentation/maturation tank at the indicated timepoints; thus, the described process reflects in-tank fermentation and cold maturation conditions prior to downstream commercial processing. After sampling, all samples were stored at −4 °C. Both the base beer and the single-addition umami-peptide beers were each prepared as three independent routine batches.

### 2.2. Instruments and Reagents

The instruments and reagents used in the experiments were listed in Table 1.

### 2.3. Experimental Procedures

#### 2.3.1. Sample Preparation

A 400 μL sample was placed in a 1.5 mL microcentrifuge tube and vortex-mixed for 30 s. It was then centrifuged at 17,000× *g* for 15 min at 20 °C. The supernatant was transferred to a new tube. Vacuum centrifugation at 35 °C was performed to dryness. The residue was reconstituted in 100 μL of 75% methanol–water, followed by vortexing for 60 s. The mixture was sonicated for 10 min. It was centrifuged again at 17,000× *g* for 10 min at 20 °C. The final supernatant was transferred to an autosampler vial for instrumental analysis.

#### 2.3.2. RPLC-Q-TOF-MS Detection and Analysis Conditions

Chromatographic conditions: A ACQUITY UPLC HSS T3 column (1.8 μm, 2.1 × 100 mm, Waters Corporation, Milford, MA, USA) was used for peptide separation. Mobile phase A was 0.10% formic acid in water. Mobile phase B was 0.1% formic acid in acetonitrile. The injection volume was 4 μL. Separation was performed with a gradient program detailed in Supplementary Table S1.

Mass spectrometric conditions: An AB SCIEX TripleTOF 5600 mass spectrometer (SCIEX, Marlborough, MA, USA) controlled by Analyst TF 1.7 control was operated in information-dependent acquisition (IDA) mode to collect MS^1^ and MS^2^ data. In each cycle, the most intense precursor ions above a 100-cps threshold were selected for MS^2^. The MS^1^ scan range was *m*/*z* 100–1500. MS^2^ acquisition used a fixed collision energy of 30 eV, with up to 10 MS^2^ spectra per cycle. ESI source parameters were: GS1 60 psi, GS2 60 psi, CUR 35 psi, temperature 550 °C, and ion spray voltage 5500 V (positive mode) [4,5,16].

#### 2.3.3. Qualitative Identification and Semi-Quantitative Analysis of Beer Peptides

Protein identification was performed with PEAKS Studio, using the search strategy described in Supplementary Table S2. The confidence threshold was set at −10logP ≥ 15. To recover peptides potentially missing from the database, the de novo sequencing function of PEAKS was applied to fragment spectra. The average local confidence (ALC, Average Local Confidence) was used to assess residue-level reliability, and only sequences with ALC ≥ 90% were retained. These de novo results complemented and cross-validated the database identifications during subsequent alignment and functional analyses [4].

Based on qualitative results, semi-quantitative peptide levels were calculated with the PVPL peptide as an internal standard. PVPL was selected as the internal standard because the present LC MS analysis focused on low-molecular-weight beer peptides, and an appropriate internal standard in peptide assays should resemble the analytes in sample preparation behavior, chromatographic retention, and ionization response while remaining analytically distinguishable from endogenous matrix signals. In peptide-based LC MS/MS methods, internal standardization is used to correct variations arising from extraction, injection, and matrix dependent ion suppression. The ideal choice is a stable isotope-labeled analog. Even so, recent methodological studies have shown that when such standards are not available or are impractical, a structurally related peptide can serve as a reasonable alternative if it provides stable response and good assay reproducibility. On this basis, PVPL was chosen because it is a short synthetic peptide within the same general molecular class as the target beer peptides, making it more suitable than a nonpeptidic compound for monitoring analytical fluctuation in a complex beer matrix. Under the present chromatographic conditions, PVPL provided a stable signal and was used to normalize signal variation among samples [1,3,8]. In the present workflow, PVPL was added directly to the beer sample before sample pretreatment at a final concentration of 10 mg/L, so that variations introduced during subsequent sample processing and instrumental analysis could be normalized more effectively. In addition, recovery was monitored under the same pretreatment procedure, and the apparent recovery of PVPL ranged from 87.92% to 137.76%. This design is consistent with current bioanalytical practice, in which the internal standard is added during sample processing and recovery is evaluated when extraction-based preparation is used. The semi-quantitative calibrant was the synthetic PVPL peptide. The standard curve was Y = 0.0039X + 6.6831 R^2^ = 0.9998.

In the present study, PVPL was used as a surrogate internal standard for signal normalization and cross-sample comparison during peptide analysis. Because PVPL is not an isotopically matched analog of the endogenous peptides detected in lager beer, it cannot fully correct for peptide-specific differences in extraction recovery, chromatographic behavior, or ionization efficiency within this complex matrix. Therefore, the PVPL-normalized peptide abundance data reported herein should be interpreted as semi-quantitative estimates only, and are mainly intended to support relative comparison of peptide abundance patterns among samples and fermentation stages rather than absolute quantification. More rigorous absolute quantification would require isotopically matched internal standards together with a fully validated quantitative workflow.

#### 2.3.4. Efficient Screening of Potential Umami Peptides Using Machine Learning

UMPred-FRL (http://pmlabstack.pythonanywhere.com/UMPred-FRL, accessed on 14 March 2026), TastePeptides-Meta (http://tastepeptides-meta.com/TPDM, accessed on 14 March 2026), and Umami-MRNN (https://umami-mrnn.herokuapp.com/) were selected because they are publicly available machine learning tools that have been previously developed and validated for umami-peptide prediction. UMPred-FRL is a feature-representation-learning meta-predictor that integrates seven sequence-based encodings, and later comparative evaluation reported independent-test performance of ACC = 0.888, MCC = 0.735, Sn = 0.786, and Sp = 0.934. TastePeptides-Meta incorporates the Umami_YYDS model, a gradient boosting decision tree classifier established through data enhancement, algorithm comparison, and model optimization, with a reported accuracy of 89.6%. Umami-MRNN was developed on the UMP-499 dataset using six feature vectors and a hybrid MLP-RNN architecture, and its independent test showed an ACC of 90.5% and an MCC of 0.811. In addition, ProUmami was used as a complementary predictor during candidate selection. Peptides were retained as high-confidence candidates only when both UMPred-FRL and ProUmami yielded prediction scores >0.9, and this stringent cutoff was adopted to reduce potential false-positive candidates during preliminary in silico screening before subsequent sensory validation. In the present study, these tools were used for preliminary in silico screening of peptides with potential umami activity, whereas the sensory thresholds and the suitability of candidates for the single-addition experiments were ultimately confirmed by sensory evaluation.

#### 2.3.5. Molecular Docking Methods

As the starting point for homology modeling, the reference sequence of the metabotropic glutamate receptor mGluR1 was obtained from UniProtKB. Its crystal structure (PDB: 1EWK, RCSB PDB) was then selected as the template. Using the amino acid sequences of the T1R1 and T1R3 subunits, a three-dimensional homology model was built on the SWISS-MODEL platform. The T1R1 and T1R3 models showed sequence identities of 34.34% and 33.55%, respectively, relative to their templates, which exceeded the empirical threshold generally considered necessary for obtaining a reliable overall fold in homology modeling. After model construction, a Ramachandran plot was generated with SAVES v6.0 to examine geometric plausibility. The plot, based on residue φ–ψ dihedral distributions, assessed conformational reliability. Ramachandran statistics showed that 97.70% of residues were located in allowed regions, including 87.70% in favored regions, 10.00% in additionally allowed regions, and 1.80% in generously allowed regions, whereas fewer than 0.5% of residues fell in disallowed regions, indicating acceptable geometric quality for subsequent docking and mechanistic analysis. In the present study, structural validation was primarily based on Ramachandran analysis; additional metrics such as PROSA or ERRAT were not further reported. After validation, the model was used for docking. Before docking, the receptor was preprocessed in PyMOL 2.6.0 to remove solvent molecules, inorganic ions, and noncovalently bound small ligands, and a docking grid covering the entire protein surface was defined. Peptide ligands were built in Discovery Studio 2019 and assigned CHARMm force-field parameters. Energy minimization was performed with Smart Minimizer (up to 2000 steps; RMS gradient threshold 0.01), and potential binding cavities were identified on the same platform. After the binding site was determined, candidates were docked into the T1R1/T1R3 complex using the semi-flexible CDOCKER protocol. Other parameters were kept at default, and only the pose with the highest CDOCKER Energy score was retained. Finally, three-dimensional visualization and interaction analysis of the complexes were carried out in PyMOL 2.6.0 and BIOVIA Discovery Studio 2019 [22,23,24,25,26,27,28].

#### 2.3.6. Umami Peptide Taste Threshold Determination

To determine recognition thresholds, TDA (taste dilution analysis) with stepwise serial dilution was employed. A stock solution with 8% (*v*/*v*) alcohol, pH 6.5, and 1 mg/mL peptide was prepared. It was diluted 1:1 with deionized water to generate a concentration series. Samples were presented to a trained sensory panel (n = 20). Panelists were selected according to general criteria for trained sensory assessors, including normal taste perception, good health status, and the ability to perform repeated discrimination tasks consistently [29,30,31,32,33]. Before formal threshold determination, all assessors underwent unified training covering basic taste recognition, familiarization with the alcohol-containing model matrix, and standardized implementation of the three-alternative forced-choice (3-AFC) procedure [29]. Samples were presented in ascending order. At each level, discrimination followed a three-alternative forced-choice (3-AFC) protocol. Each presentation contained one sample and two blanks. Panelists identified the odd sample and recorded the lowest recognizable concentration. The set was then repeated for confirmation. Panel performance and response repeatability were preliminarily validated through duplicate threshold determinations, and only consistent judgments obtained across repeated evaluations were used for threshold assignment. This duplicate-confirmation step served as an internal check of assessor consistency and threshold reliability. When the two trials agreed, the value was taken as the threshold.

#### 2.3.7. Molecular Dynamics (MD) and MM/GBSA Binding Free Energy Calculation

Docking-derived peptide–receptor complexes were used as initial conformations. All-atom MD simulations were performed with AMBER 22. The protein and peptides were parameterized with ff14SB. Hydrogens were added in LEaP. A truncated octahedral TIP3P water box was built with a 10 Å minimum solute–boundary distance. Na^+^/Cl^−^ counterions were added to neutralize the system, and topology and coordinate files were generated. Energy minimization used 2500 steps of steepest descent followed by 2500 steps of conjugate gradient. The system was heated under NVT for 200 ps from 0 K to 298.15 K. A further 500 ps NVT run equilibrated solvent distribution. Pre-equilibration then proceeded under NPT for 500 ps. Production was carried out for 100 ns under NPT with periodic boundary conditions. The nonbonded cutoff was 10 Å. Long-range electrostatics were treated with particle–mesh Ewald. Bonds involving hydrogens were constrained with SHAKE. Temperature was controlled by Langevin dynamics (γ = 2 ps^−1^). Pressure was maintained at 1 atm. The time step was 2 fs. Trajectories were saved every 10 ps for analysis [23,34,35,36,37,38,39]. Trajectory processing and structural analysis were performed using CPPTRAJ v6.4.4 in AmberTools 22. Structural stability was evaluated from the MD trajectories, and the equilibrated portion of the simulation was used for subsequent free-energy analysis. Each docking-derived peptide–receptor complex was subjected to one 100 ns production MD simulation, and replicate independent production runs initiated with different random velocities were not performed. The MM/GBSA calculations were therefore based on a single-trajectory workflow for each complex. The 90–100 ns interval was not selected merely because it represented the terminal part of the simulation, but because trajectory inspection indicated that the major structural descriptors monitored in this work had entered a comparatively stable fluctuation regime during this late stage, with no obvious persistent drift relative to the preceding portion of the production run. Accordingly, this time window was operationally defined as the most stable segment available for end-point free-energy evaluation and residue-wise energy decomposition. At the same time, because this assessment was derived from one continuous trajectory per complex rather than from replicate simulations, the selected 90–100 ns window should be regarded as an empirically stabilized analysis interval within the sampled trajectory, rather than as definitive proof of full conformational convergence.

Binding free energies were calculated within the MM/GBSA framework. To reduce time correlation and conformational drift, trajectory segments from the 90–100 ns window were used for statistical analysis. This time window corresponded to 1000 saved frames and was treated as the converged segment for energy evaluation. For each complex, the binding free energy was summarized as the mean ± standard deviation over the selected frames, and the same trajectory window was used for per-residue energy decomposition. The total free energy was decomposed as follows:

(1) $$\Delta G_{bind}={\Delta G}_{\mathrm{complex}}-({\Delta G}_{\mathrm{receptor}}+\cdot {\Delta G}_{\mathrm{ligand}})={\Delta E}_{\mathrm{internal}}+{\Delta E}_{\mathrm{vDW}}+{\Delta E}_{\mathrm{elec}}+{\Delta G}_{\mathrm{GB}}+{\Delta G}_{\mathrm{SA}}$$

In Equation (1), ΔE_internal_ denoted internal energy, ΔE_vdw_ denoted van der Waals interactions, and ΔE_elec_ denoted electrostatic interactions. Internal energy comprised bond (E_bond_), angle (E_angle_), and torsional (E_torsion_) terms. ΔG_GB_ and ΔG_SA_ were collectively referred to as the solvation free energy. Here, G_GB_ represented the polar solvation component, and G_SA_ the nonpolar component. For ΔG_GB_, the GB model developed by Nguyen et al. was used (igb = 2). The nonpolar solvation free energy (ΔG_SA_) was calculated as the product of the surface tension (γ) and the solvent-accessible surface area (SA), as in

(2) $$\Delta G_{SA}=\cdot 0.0072\cdot \times \cdot \Delta \mathrm{SASA}$$

Entropy changes were neglected due to high computational cost and limited accuracy [40,41,42].

#### 2.3.8. Sensory Evaluation and Single-Addition Variable Method

Sample codes and volumes were as follows: 20 mL of lager beer were accurately measured from fermentation day 0, 1, 3, 10, 17, and 29. The original samples were labeled A, B, C, D, E, and F. After single addition of umami peptides (ATTSIA, TVDVS, ATTSI, ATTSL, RSEQ, ATSTLA), the samples were labeled A-1–A-6, B-1–B-6, C-1–C-6, D-1–D-6, E-1–E-6, and F-1–F-6. The amount of each single addition was 500 μL of the threshold solution. The threshold solutions used for single addition were determined previously by the 3-alternative forced-choice (3-AFC) method. In brief, each 3-AFC set consisted of three coded samples, including one peptide-containing sample and two corresponding blank controls, and assessors were required to identify the odd sample; the test was conducted in ascending concentration order to determine the threshold concentration for each peptide. First, samples A–F were evaluated sensorially. Sensory evaluation was performed by a trained panel of 20 assessors. Panel training covered the definition, recognition, and scale use of the target beer sensory attributes, and specific screening for umami sensitivity was incorporated into this training process rather than conducted as a separate standalone test. Specifically, umami sensitivity was assessed during training using reference-guided recognition exercises with graded umami standard solutions, and only assessors who could correctly recognize the umami attribute and show stable scoring performance were admitted to the formal evaluation. Panel consistency was verified before formal evaluation by triangle-test-based training and qualification. Descriptions of the sensory dimensions and scoring criteria were provided in Supplementary Table S3, which presented nine key attributes on a 0–9 scale to standardize assessment of aroma, taste, and mouthfeel [43,44,45,46,47,48]. The anchors of the scale ranged from 0 (not perceptible) to 9 (extremely intense), and assessors scored samples according to the predefined attribute descriptions and reference-guided training framework. Thereafter, the same sensory scoring was applied to A-1–A-6, B-1–B-6, C-1–C-6, D-1–D-6, E-1–E-6, and F-1–F-6, and changes in beer sensory properties after single addition were compared. All samples were evaluated blind, coded with random three-digit numbers, and presented monadically in randomized order under red light to minimize visual and order bias. Each sample was evaluated in triplicate, but the three replicates were presented in separate randomized rounds rather than as immediate consecutive repeats to reduce adaptation and memory effects. Carryover and sensory fatigue were managed by palate cleansing with water and unsalted crackers between samples, together with short rest intervals between evaluation blocks. Each sample was presented in triplicate, and repeated sensory assessments were conducted to improve data robustness. Carryover was managed by providing water and unsalted crackers as palate cleansers between samples. Samples were served at 8 ± 1 °C. For each attribute, sensory scores were summarized as mean ± standard deviation across panelists. Because sensory comparisons were made either across fermentation-stage beer samples or between peptide-added samples and their corresponding stage-matched controls, the beer matrix was kept consistent within each comparison set; thus, ethanol background and carbonation were controlled by using the original beer sample of the same stage as the direct matrix reference. Because the experimental design involved one factor with multiple sample levels, one-way ANOVA was used to test overall differences in sensory scores among fermentation stages and among peptide-addition groups. The resulting sensory matrix was then used for subsequent correlation analysis, heat-map visualization, and cluster analysis.

#### 2.3.9. Statistical Analysis

Radar and fingerprint plots were generated in OriginPro 2021 (OriginLab Corporation, Northampton, MA, USA) to visualize multivariate profiles. Heat maps were produced using TBtools-II v2.303 (South China Agricultural University, Guangzhou, China). One-way analysis of variance (ANOVA) and correlation analyses were performed using IBM SPSS Statistics 24.0 (IBM Corp., Armonk, NY, USA). Sensory data are presented as mean ± standard deviation. One-way ANOVA was used to compare mean values of individual sensory attributes among sample groups, because the design involved a single experimental factor with multiple levels. Pearson correlation analysis was used to assess linear relationships between peptide-related variables and sensory attributes. Statistical significance was set at *p* < 0.05, corresponding to a 95% confidence level, and correlation coefficients with |r| > 0.70 were considered to indicate a strong correlation. Cluster analysis was conducted in R v4.3.2 (R Foundation for Statistical Computing, Vienna, Austria), using the hclust function based on Euclidean distance and the Ward.D2 linkage method, and the clustering results were extracted and visualized within the R environment.

## 3. Results and Analysis

### 3.1. Qualitative Identification of Peptides in Lager Beer

Database-matched peptides were filtered with a confidence threshold of −10logP ≥ 15. For de novo results, only sequences with ALC ≥ 90% were retained to ensure reliability. Following this workflow, 1741, 2395, 1904, 2079, 3716, and 3515 peptides were obtained at fermentation days 0, 1, 3, 10, 17, and 29, respectively. Umami attributes were then predicted using UMPred-FRL and TastePeptides-Meta. Qualitative information and umami predictions for peptides in the lager matrix were provided in Supplementary Tables S4–S9.

Patterns were derived from the peptide distributions in Supplementary Tables S4–S9 and from a review of the brewing fermentation literature. Across days 0, 1, 3, 10, 17, and 29, the numbers of identified peptides were 1741, 2395, 1904, 2079, 3716, and 3515, respectively. When the detected sequences were compared as day-specific peptide sets, peptide length, estimated molecular weight, and GRAVY hydrophobicity index all differed significantly among fermentation days (Kruskal–Wallis, all *p* < 0.001), and the distribution of short peptides (≤5 aa), medium peptides (6–10 aa), and long peptides (≥11 aa) also changed significantly over time (χ^2^ = 331.36, *p* < 0.001). On day 0, the peptide pool was dominated by short soluble sequences, with peptides of ≤5 aa accounting for 87.8%, a mean peptide length of 4.20 aa, a mean estimated molecular weight of 459.0 Da, and a mean GRAVY value of 0.75. This pattern is consistent with the view that wort provides an initial nitrogen reservoir composed of FAN together with readily soluble peptides generated during malting and mashing [49,50]. On day 1, the total peptide number increased to 2395, while the mean peptide length rose slightly to 4.65 aa and the proportion of aromatic-residue-containing peptides reached 31.5%, the highest level among the early stages. This result suggests that early after inoculation, yeast rapidly consumed FAN and part of the most readily assimilable small peptides, while extracellular or periplasmic peptidase activity continued to reshape the soluble peptide pool [51,52,53,54,55]. By day 3, the peptide count decreased to 1904, but the mean peptide length increased markedly to 5.60 aa, the proportion of 6–10-residue peptides increased to 25.9%, and the proportion of peptides containing acidic residues rose to 33.5%. Thus, the day 3 peptide profile reflected not a simple quantitative loss, but a selective remodeling process in which very short readily assimilable peptides were depleted more rapidly, whereas longer and relatively less hydrophobic sequences became proportionally enriched. By day 10, the total number recovered modestly to 2079, but the peptide pool still differed clearly from wort, showing a mean length of 5.41 aa, a mean GRAVY value reduced to 0.58, and continued enrichment of 6–10-residue peptides. Taken together, the day 3 and day 10 data are more consistent with ongoing peptide redistribution under fermentation acidification, CO_2_ accumulation, yeast flocculation, and colloid-associated removal than with uniform peptide accumulation, especially because protein-polyphenol interactions in beer are known to be initially reversible and then to promote aggregation and sedimentation over time [56,57].

From day 10 to day 17, the peptide count increased sharply from 2079 to 3716, representing a 78.7% rise, and remained high at 3515 on day 29. In parallel, the average peptide profile became distinctly less hydrophobic. The mean GRAVY value fell from 0.58 on day 10 to 0.21 on day 17 and 0.23 on day 29, while the proportion of peptides containing acidic residues increased to 37.9% and 35.9%, respectively, which were the highest values observed across the full fermentation process. Although peptides of ≤5 aa still predominated at these two lagering stages, accounting for 76.9% and 75.5%, the average peptide length stabilized near 5 aa, and no significant difference was observed between days 17 and 29 in peptide-length distribution or GRAVY distribution (both *p* > 0.05). This indicates that mid-to-late fermentation was characterized less by abrupt directional turnover and more by the establishment of a quasi-steady peptide state. Notably, only 73 peptides were shared across all 6 fermentation days, whereas the numbers of day-specific peptides were 744, 1148, 894, 983, 2556, and 2405 for days 0, 1, 3, 10, 17, and 29, respectively, further demonstrating the strong stage dependence of peptide composition. Mechanistically, the late rise in peptide number together with the shift toward lower hydrophobicity is consistent with mild yeast autolysis and the continued release of intracellular and cell-wall-associated peptide material, while hydrophobic and haze-active sequences remained more susceptible to colloidal association and removal [58,59]. Therefore, the peptide profile of this lager system did not evolve linearly. Rather, it progressed from a wort-stage pool enriched in short soluble peptides, to an early-fermentation pool undergoing selective remodeling, and finally to a late-lagering state characterized by higher peptide diversity, lower average hydrophobicity, and enrichment of low- to medium-molecular-weight peptides.

In summary, the peptide evolution observed in this lager system should be interpreted as a statistically supported process of sequential restructuring rather than as a simple increase or decrease in peptide number. Although the total number of detected peptides fluctuated across fermentation, multivariate analysis showed that the overall peptide profile followed a clear stage-dependent trajectory, with early fermentation, middle fermentation, and late maturation forming distinguishable compositional states. In parallel, the relative representation of peptide classes changed significantly over time, indicating that the final peptide pool was shaped by selective retention and removal, not merely by cumulative peptide release [57,58].

From the perspective of screening relevance, peptide importance was defined more rigorously by combining temporal recurrence, structural plausibility, and later sensory verification [59,60]. In other words, peptides detected repeatedly across key fermentation stages were considered more meaningful than peptides appearing only transiently at a single timepoint, because recurrent occurrence better reflects process stability and reduces the likelihood of stage-specific analytical noise. This criterion was further strengthened by the observation that the late-stage peptide pool was relatively enriched in low- to medium-molecular-weight, less hydrophobic, and more acid-residue-containing sequences, which are more consistent with the structural characteristics commonly associated with umami peptides [61,62,63,64]. The requirement for detection in at least two key stages was adopted as a pragmatic minimum-recurrence threshold rather than an arbitrary cutoff. Specifically, a one-stage criterion would be more susceptible to stochastic MS sampling and stage-specific analytical noise, whereas a more stringent requirement, such as persistence across three or more stages, could exclude low-abundance but potentially relevant peptides whose generation was temporally restricted during fermentation. Thus, occurrence in two stages was used here as evidence of basic temporal reproducibility while preserving peptide diversity for downstream validation, which is consistent with LC-MS data-filtering strategies that aim to remove uninformative features while retaining biologically meaningful signals, as well as with proteomic workflows that commonly retain features or identifications observed in at least two replicates for subsequent analysis. Accordingly, Section 3.1 supports the view that fermentation did not produce a random peptide background. Instead, it progressively selected a more flavor-relevant peptide subset, thereby providing a clearer molecular basis for subsequent candidate screening, receptor-level validation, and the interpretation of umami perception in lager beer.

### 3.2. Preliminary Screening of Umami Peptides and Molecular Docking Analysis

**Figure 1:**
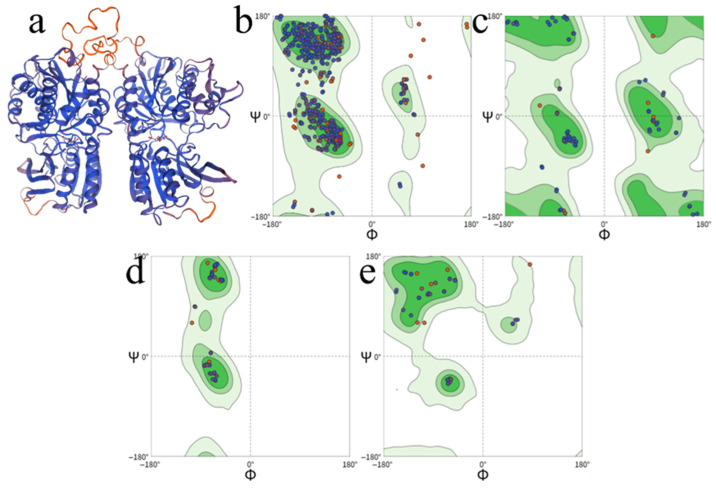
Homology modeling results of the umami receptor (**a**) Homology-modeled structure of T1R1/T1R3; Ramachandran plots: (**b**) general, (**c**) proline, (**d**) glycine, (**e**) pre-proline.

The dimer was split into two subunits, homology models were built separately, and geometric plausibility was examined with a Ramachandran plot [65,66,67]. As shown in Figure 1a, T1R1 adopted a relatively closed conformation, whereas T1R3 displayed a more open conformation, forming a cavity large enough to accommodate long-chain umami peptides. Regarding the binding site, existing evidence supported interactions of ligands with the VFT domains of both subunits. T1R1 was critical for recognizing amino acids and short peptides, and T1R3 acted cooperatively in ligand recognition and conformational regulation. Both chains are therefore indispensable for taste transduction. For homology modeling, usable folds and overall topology were generally obtained when target–template identity was ≥30%. In this study, the identities of the T1R1 and T1R3 models to their templates were 34.34% and 33.55%, meeting this empirical threshold. Further Ramachandran statistics (Figure 1b–e) showed that 97.70% of residues fell in allowed regions, with 87.70% in favored regions, 10.00% in additionally allowed regions, 1.80% in generously allowed regions, and <0.5% in disallowed regions. More than 90% of φ–ψ angles lay within reasonable ranges, indicating geometric quality adequate for subsequent docking and mechanistic analysis. Based on the above screening criteria, candidates with both UMPred-FRL probability and ProUmami score > 0.9 were selected. In total, 112 low-molecular-weight peptides were advanced to docking with the T1R1/T1R3 receptor (Table 2). The T1R1/T1R3 receptor was a class C GPCR heterodimer. Its extracellular Venus flytrap (VFT) ligand-binding domain undertook recognition and fixation of umami ligands. Molecular docking was performed with the semi-flexible CDOCKER protocol in Discovery Studio (DS). Other parameters were kept at default, and only the pose with the lowest docking energy was retained. In the end, 86 peptides docked successfully (Table 2), including 1 dipeptide, 25 tripeptides, 31 tetrapeptides, 16 pentapeptides, and 13 hexapeptides.

Peptides that docked successfully exhibited relatively consistent physicochemical and sequence features. Their lengths concentrated in di- to hexapeptides, mostly tri- and tetrapeptides, which aligned with the conformational constraints of the T1R1/T1R3 extracellular VFT domain that favored small ligands. Acidic residues (E/D) coexisted with small hydrophobic/neutral residues (A/T/V/S/L). Common short motifs included “E–” and “…TSI/TSL/TTY/TV…”. These provided salt-bridge and hydrogen-bond anchors, while hydrophobic side chains stabilized the pocket microenvironment. Strongly basic and sulfur-containing reactive residues were rare (only sporadic K/C). The overall charge was mild, and the conformation was flexible. These patterns were consistent with empirical observations that umami peptides were enriched in acidic residues and carried hydrophobic/neutral residues at termini, and with the T1R1/T1R3 recognition mechanism for small peptides/amino acids in which the VFT pocket selected ligand size and charge distribution. By contrast, peptides that failed to dock were longer overall, including 8–22-mers, and showed higher proportions of Cys/Arg/Pro or multiple hydrophobic blocks. Such sequences tended to introduce excessive conformational freedom or spontaneous folding penalties, and caused steric hindrance at the pocket entrance or unfavorable solvation–desolvation costs. When an N-terminal acidic anchor or an arrangement capable of forming a stable hydrogen-bond network was lacking, insufficient induced fit with unfavorable docking energy was more likely. It should be emphasized that docking failure was not determined by a single factor. A few short peptides (e.g., EVG, AAGIE) also failed, indicating that charge orientation, side-chain geometry, and micro-motifs were equally critical. These findings agreed with recent peptide library screening and molecular simulations targeting T1R1-VFT, showing that bindable or activatable peptides constituted a subset defined by specific residue patterns and spatial orientations rather than arbitrary short peptides.

From the perspective of fermentation time, multiple tri- to hexapeptides were already detected at 0 d (wort), such as VGIT/TIE/EVA/ELT/ENM/TTY and AFTPLQ/QLSESE/ATTSIA/LDVATD. These findings suggested that malt proteases during saccharification had released initial fragments containing acidic residues (E/D) with potential umami phenotypes. At 1 d, new short peptides appeared that were enriched in E/D and terminated with hydrophobic residues such as V/A/L (e.g., EAV, ESY, ELE, VEY, EGLA, IEVVD, AAEVLE), reflecting initial cleavage and terminal trimming by yeast-secreted endo-/exopeptidases. By 3 d, transitional tetra- to hexapeptides and “ATTS/TV DVS” micro-motifs (TVSGF, LEDI, AATIQ, ATTSLA, TVDVSA, RSEQ) emerged, indicating endo–exo synergy that further regularized fragments and drove enrichment toward specific sequence scaffolds. At 10 d, families expanded with characteristic termini such as Q/T/A–T–T/S– and E/D–V (QCCQQ, TVLT, EIVDV, DIVATD, TATY, EAVT, MAVTGF), and the lineage shifted toward shorter length with higher E/D content. At 17 d, both peptide numbers and types increased markedly, and multiple variants appeared as positional swaps or terminal pairs (e.g., GVVT/VSGV, TVGE/TVES, EPEP/TRST, VDYGG/TMPT), indicating “family-like” expansion driven by bidirectional exopeptidase processing and yeast autolysis. In the late fermentation and maturation stage (29 d), the profile reached a peak. Multi-site variants of the ATTS/TATS/VT(S)GF families were detected (ATTSI/ATTSL/ATSTLA/TATSLA/TATSI/TATSL/VTSGF). Shorter di-/tripeptides also appeared (EM, EVQ, NDT, YTS, VTY), together with mirror/symmetric sequences (e.g., VEEV, GVTV/VTGV, LSVP corresponding to earlier LSVE). These features reflected ongoing hydrolysis during late fermentation–maturation–yeast autolysis that enriched short, E/D-rich peptides and reshaped the profile. Overall, a continuous temporal trajectory was observed: initial release in wort, structural refinement in early–mid stages, late-stage family diversification with a shift toward shorter length.

Taken together, umami peptides were nonvolatile polypeptides, and their perception was mediated mainly by taste receptors (T1R1/T1R3). Their perception exhibited a “cluster/family-style activation,” in which multiple short peptides with similar sequences formed an interchangeable binding network with the receptor pocket through shared acidic–hydrophobic motifs, showing group substitutability and temporal robustness. This view was compatible with the VFT dual-site cooperativity and ligand–pocket multiconformation models and was consistent with statistical findings that umami peptides were enriched in acidic residues and displayed specific sequence patterns. Therefore, representative umami peptides were selected for molecular simulation and sensory validation to characterize the common activation features of this class of peptides.

Based on the above descriptions and the docking results in Table 2, six potential umami peptides—ATTSIA, TVDVS, ATTSI, ATTSL, RSEQ, and ATSTLA—were selected. Their selection was based on a comprehensive strategy rather than single-factor screening. First, all six peptides were positively retained after the in silico workflow and were able to generate acceptable binding poses in the T1R1/T1R3 receptor model, indicating that they met the basic requirement of receptor recognition plausibility for subsequent validation. Second, these peptides fall within a short-chain range of 4–6 amino acid residues, which is consistent with the size characteristics commonly reported for many taste-active umami peptides and is advantageous for receptor access, peptide synthesis, and threshold determination. Third, the final set was intentionally designed to preserve sequence diversity rather than repeatedly selecting only one structural type. Among them, TVDVS and RSEQ contain charged residues and represent peptides with relatively stronger electrostatic-interaction potential, whereas ATTSIA, ATTSI, ATTSL, and ATSTLA are Ala/Thr/Ser-rich neutral short peptides, representing another major structural type in which hydrogen bonding, hydrophilicity, and subtle residue substitution may play dominant roles in receptor recognition. This distinction was important because recent studies have shown that umami peptide binding is not governed by one universal motif only, but by the combined effects of peptide length, amino acid composition, hydrophilicity, hydrogen-bonding pattern, and the participation of residues such as Asp, Glu, Ala, Gly, Ser, and Thr in T1R1/T1R3 recognition. Fourth, temporal representativeness in the fermentation process was also considered. TVDVS was detected on days 0, 10, and 29, ATTSI and ATTSL on days 0, 3, and 29, and ATSTLA on days 0 and 29, indicating that these peptides were not restricted to a single transient stage but persisted across key process nodes. Such recurrence increased their value as candidate peptides because it suggested that they were more likely to reflect stable process-related peptide generation than accidental or highly transient sequence appearance. In contrast, ATTSIA and RSEQ were retained as complementary candidates despite being stage-specific, because they expanded the structural and physicochemical coverage of the final test set and displayed favorable receptor-binding behavior, thereby preventing the candidate pool from being biased only toward recurrent peptides. Fifth, the four homologous ATT(S)-containing peptides were deliberately retained as a compact structure–activity subseries. ATTSI and ATTSL differ only in the terminal Ile/Leu substitution, whereas ATTSIA and ATSTLA introduce terminal extension and residue rearrangement on a closely related backbone. Keeping these homologous peptides in the final set allowed subsequent experiments to evaluate whether very small sequence variations could lead to measurable differences in binding stability, taste threshold, and sensory contribution. This was scientifically important because one of the central questions in umami-peptide research is how subtle sequence changes modulate receptor interaction and final taste expression. Therefore, the six selected peptides were considered a balanced subset that combined favorable receptor-binding plausibility, structural diversity, process representativeness, comparative value for structure–activity analysis, and practical suitability for downstream synthesis and sensory verification. In this way, the final six peptides were not merely the top-scoring docking outputs, but representative candidates selected to balance receptor-level plausibility, temporal occurrence, sequence diversity, and experimental tractability. Subsequent work included peptide synthesis, taste threshold determination (Supplementary Table S10), molecular dynamics simulations, and single-addition variable experiments.

### 3.3. Analysis of Binding Modes Between Six Umami Peptides and the Receptor Protein

For comparative interpretation, L-glutamate, the canonical orthosteric ligand of the human T1R1/T1R3 umami receptor, was included as a reference ligand and docked into the same VFT binding region under the same protocol. Therefore, the binding modes of the six peptides were interpreted relative to a known umami ligand rather than as isolated docking outcomes. In this context, glutamate served as a structural reference for receptor recognition plausibility, whereas the six peptides were evaluated as candidate ligands with potentially different binding patterns and stabilizing interactions. Overall, the six short peptides (ATTSIA, TVDVS, ATTSI, ATTSL, RSEQ, ATSTLA) adopted an inter-subunit bridging mode. The ligands inserted into the narrow cleft at the junction of the extracellular VFT domains of T1R1–T1R3 and were stabilized at the dimer interface. This spatial placement aligned with the structure–function consensus that T1R1/T1R3 was a class C GPCR heterodimer whose primary ligand site resided in the VFT.

**Figure 2:**
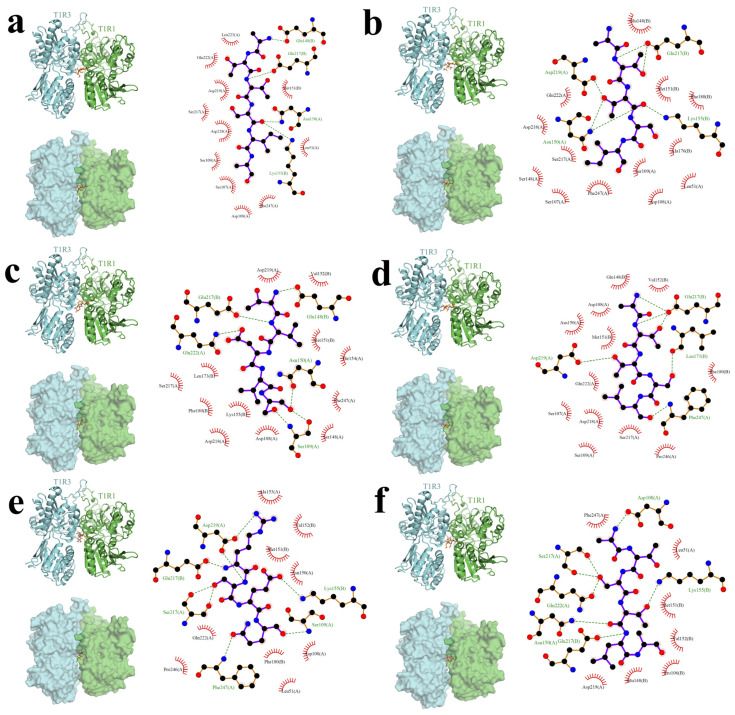
Schematic of molecular docking Left panel shows the overall view: the peptide ligand is displayed as an orange stick, T1R1 is colored green, and T1R3 is colored cyan. Right panel shows the 2D interaction map, where dashed lines indicate hydrogen bonds; chain A corresponds to T1R1 and chain B to T1R3. (**a**) binding mode of T1R1–T1R3/ATTSIA obtained from docking; (**b**) binding mode of T1R1–T1R3/TVDVS obtained from docking; (**c**) binding mode of T1R1–T1R3/ATTSI obtained from docking; (**d**) binding mode of T1R1–T1R3/ATTSL obtained from docking; (**e**) binding mode of T1R1–T1R3/RSEQ obtained from docking; (**f**) binding mode of T1R1–T1R3/ATSTLA obtained from docking.

As shown in Figure 2a, ATTSIA, displayed as an orange stick, was embedded in the narrow pocket formed between the T1R1 and T1R3 subunits and bridged their interface. In the 2D interaction map, stable contacts were formed with key residues. Hydrogen bonds were observed with Glu217(B), Asp219(A), Asn150(A), Glu148(B), and Lys155(B), which stabilized the ligand conformation at the active site. The region surrounding the pocket contained many residues contributing hydrophobic interactions, including Met151(B), Phe180(B), Val176(B), Leu51(A), Phe247(A), Ser109(A), and Asp108(A), which further reinforced the binding mode.

As shown in Figure 2b, TVDVS was embedded in the narrow pocket between T1R1 and T1R3 and bridged the interface. In the 2D interaction map, stable contacts were formed with several key residues. Multiple hydrogen bonds and hydrophobic interactions stabilized the complex. Hydrogen bonds mainly involved Glu217(B), Gln222(A), Glu148(B), Asn150(A), and Ser109(A). These bonds fixed the peptide conformation and enhanced binding stability. The region around the pocket contained numerous residues that strengthened binding through hydrophobic effects, including Asp219(A), Asp218(A), Asp108(A), Ser148(A), Phe247(A), Thr154(A), Met151(B), Val152(B), Leu173(B), Phe180(B), and Lys155(B).

As shown in Figure 2c, ATTSI was embedded in the narrow pocket between T1R1 and T1R3 and bridged the interface. In the 2D interaction map, stable contacts were formed with key residues. Hydrogen bonds were observed with Glu217(B), Asp219(A), Asn150(A), Glu148(B), and Lys155(B), which stabilized the ligand conformation at the active site. The binding region contained many residues contributing hydrophobic interactions, including Met151(B), Phe180(B), Val176(B), Leu51(A), Phe247(A), Ser109(A), and Asp108(A), which further strengthened the binding mode.

As shown in Figure 2d, ATTSL was embedded in the narrow pocket between T1R1 and T1R3 and bridged the interface. In the 2D interaction map, stable contacts were formed with multiple key residues. The ligand formed a hydrogen-bond network with Asp219(A), Glu217(B), Leu173(B), and Phe247(A), which enhanced anchoring at the binding site. The surrounding region contained many residues that contributed hydrophobic interactions, such as Glu148(B), Val152(B), Met151(B), Phe180(B), Asp108(A), Asp218(A), Ser109(A), Ser217(A), and Pro246(A), further stabilizing the complex.

As shown in Figure 2e, RSEQ, was embedded in the narrow pocket between T1R1 and T1R3 and bridged the interface. In the 2D interaction map, tight interactions were formed with key residues. Hydrogen bonds mainly involved Asp219(A), Glu217(B), Ser217(A), Ser109(A), Phe247(A), and Lys155(B). These bonds formed a stable interaction network and maintained the ligand conformation at the binding site. The region around the pocket contained many hydrophobic contributors, including Met151(B), Val152(B), Ala153(A), Phe180(B), Asp108(A), Leu51(A), Gln222(A), and Pro246(A), which increased binding tightness and stability.

As shown in Figure 2f, ATSTLA, was embedded in the narrow pocket between T1R1 and T1R3 and bridged the interface. In the 2D interaction map, stable contacts were formed with several key residues. Hydrogen bonds mainly occurred with Glu148(B), Lys155(B), Asn150(A), and Phe247(A), which enhanced binding stability at the active site. Multiple residues were positioned to contribute hydrophobic interactions around the ligand, including Pro106(B), Val152(B), Met151(B), Leu173(B), Thr179(B), Ser109(A), Pro246(A), and Asp218(A), further consolidating the binding mode.

At the micro-interaction level, all six peptides exhibited a binary stabilization mechanism of polar anchoring plus hydrophobic clamping. First, ligands formed multipoint hydrogen-bond/salt-bridge networks with polar sites; recurrent anchors across conformations involved residue clusters Glu217(B), Asp219(A), Glu148(B), Asn150(A), Ser109(A), Lys155(B), and Phe247(A), which locked ligand conformation and position. Second, a hydrophobic belt around the pocket—Met151, Val152, Phe180, Leu173, Val176, Leu51, and Pro246—provided hydrophobic shielding and desolvation compensation, jointly increasing residence time and binding tightness.

Sequence-dependent differences were also evident. TVDVS and RSEQ carried D/E acidic side chains, which formed strong electrostatic coordination and stable hydrogen-bond clusters in the polar VFT pocket, suggesting higher affinity and tighter conformational constraint. ATTSIA, ATTSI, ATTSL, and ATSTLA lacked D/E, yet built alternative hydrogen-bond–hydrophobic cooperative networks via S/T residues with local terminal hydrophobes (I/L); among them, ATTSL and ATSTLA showed more continuous hydrogen-bond paths and more complete hydrophobic burial. Overall, the six peptides shared a receptor feature of interface-bridging, multivalent, low-specificity binding, while affinity and stability were co-modulated by acidic-anchor density, terminal hydrophobic exposure, and hydrogen-bond accessibility. These observations were consistent with views that VFT pockets use multiple sites with low specificity that can be fine-tuned by sequence motifs [68,69,70,71,72,73,74]. On this basis, molecular dynamics simulations were then performed.

It should also be noted that the present docking analysis provided in silico predictions of receptor recognition behavior rather than direct experimental proof of umami activity. In the current study, the docking results were used mainly for comparative screening and structural interpretation, and the calculated binding modes or interaction energies should not be overinterpreted as direct evidence that a peptide is an authentic umami ligand in vivo or that it will necessarily elicit umami perception by itself. Although glutamate was included as a reference ligand to improve interpretability, this comparison remained computational in nature. In addition, receptor-level reproducibility was controlled here by applying the same receptor model, binding region, and docking settings to all ligands, but formal protocol validation procedures such as redocking, RMSD-based pose recovery, and experimental receptor assays were not included in this section. Therefore, the present results should be interpreted as structural support for candidate prioritization, whereas the final taste relevance of these peptides should rely on the subsequent threshold determination, molecular dynamics analysis, and single-addition sensory validation presented in later sections. Further receptor-based biological assays and more rigorous docking validation would strengthen the mechanistic certainty of this part of the study.

### 3.4. Molecular Dynamics Simulation Analysis

#### 3.4.1. Stability Analysis

To strengthen the interpretation of the MD results, convergence was evaluated here using the joint behavior of ligand RMSD, complex RMSD, protein RMSF, Rg, hydrogen-bond number, and SASA rather than any single descriptor alone. In the absence of independent replicate trajectories, each 100 ns simulation was treated as a comparative stability screen performed under the same force field, solvent model, equilibration protocol, and trajectory analysis workflow [23,34,35,36,37,38,39]. Therefore, the results below are interpreted comparatively and semiquantitatively. A trajectory was regarded as reasonably converged when the ligand and complex RMSD entered a bounded plateau after the initial relaxation stage, when Rg remained within a narrow fluctuation window without progressive drift, and when the largest RMSF peaks were restricted mainly to distal loop or terminal regions rather than the binding core.

**Figure 3:**
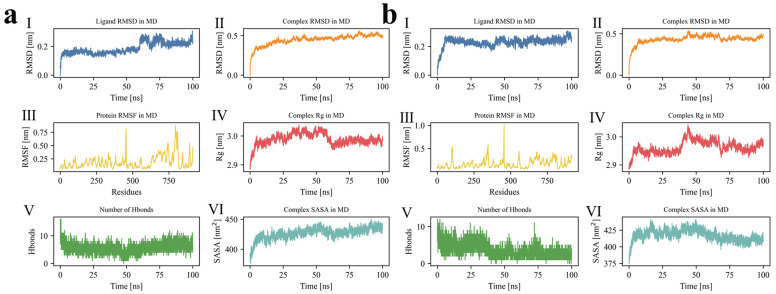

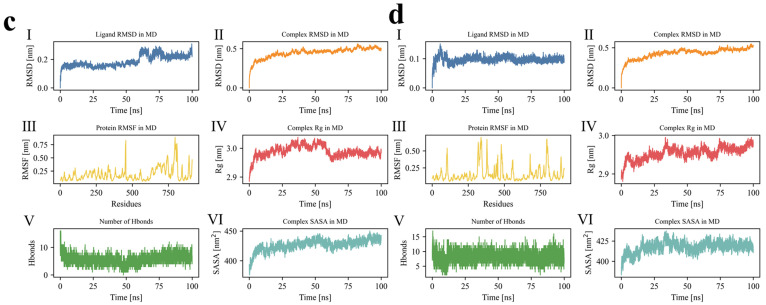

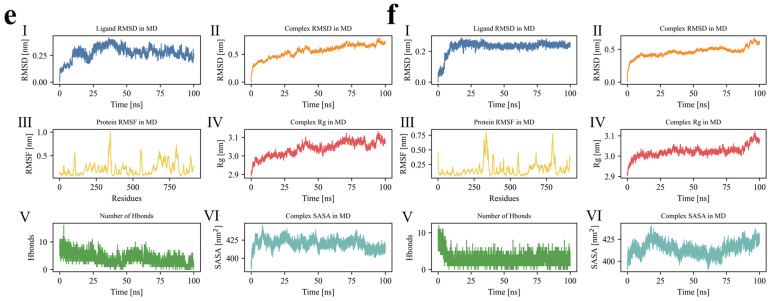
Stability analysis of binding between umami peptides ATTSIA (**a**), TVDVS (**b**), ATTSI (**c**), ATTSL (**d**), RSEQ (**e**), ATSTLA (**f**) and the T1R1–T1R3 umami receptor. Note: (**I**) ligand RMSD in MD; (**II**) complex RMSD in MD; (**III**) protein RMSF in MD; (**IV**) complex radius of gyration (Rg) in MD; (**V**) number of H-bonds; (**VI**) complex solvent-accessible surface area (SASA) in MD. All metrics were plotted against simulation time.

As shown in Figure 3a, ATTSIA adapted rapidly at the start of the simulation. Its RMSD rose to ~0.2 nm and remained largely confined to 0.20–0.25 nm for most of the production stage, indicating that only limited conformational deviation occurred after initial accommodation in the binding pocket. After ~70 ns, slight fluctuations approached ~0.25 nm, suggesting minor late-stage local readjustment rather than loss of the bound pose. The complex RMSD increased to ~0.45 nm within the first 20 ns and then drifted gently to ~0.5 nm over the run. Because this increase was followed by a broad plateau rather than continued divergence, the trajectory is more consistent with early structural relaxation and subsequent dynamic equilibration than with ongoing instability. Protein RMSF values were generally steady. Core residues mostly ranged from 0.1 to 0.3 nm, with peaks near the C terminus (~residue 800) and in several loop regions (~residues 500 and 700) approaching ~0.7 nm. These higher-amplitude fluctuations were spatially localized and did not coincide with the principal binding region, supporting the view that the flexible response was peripheral rather than disruptive to the peptide-binding core. The radius of gyration (Rg) increased from ~2.89 to ~3.0 nm, with a clearer rise after ~60 ns. This change corresponded to an increase of only ~0.11 nm, which indicates modest global relaxation while preserving the overall compact fold of the complex. Hydrogen-bond counts were ~10–13 during 0–50 ns, evidencing an early stable H-bond network. After 50 ns the count decreased to ~5–8 and stayed at that level, implying local reorganization or partial contact loss. Because the later hydrogen-bond population remained stable rather than collapsing toward zero, the data support interfacial rearrangement to a new equilibrium contact pattern rather than peptide dissociation. SASA rose from ~400 to ~425 nm^2^, indicating progressive surface exposure after binding. Taken together, the synchronized late increase in Rg and SASA, together with a stabilized but reduced hydrogen-bond network, suggests adaptive interface remodeling under retained global stability.

As shown in Figure 3b, the RMSD of TVDVS rose to ~0.22 nm within the first 10 ns, then fluctuated narrowly between 0.20 and 0.25 nm, indicating good conformational stability. The narrow ligand RMSD window indicates that once bound, TVDVS sampled only a limited conformational space, which is consistent with a well-retained pose. No late increase or large perturbation was observed, suggesting a fixed conformation and stable positioning at the binding site. The complex RMSD increased rapidly during the first 25 ns and then approached a plateau, fluctuating within 0.48–0.52 nm. The post-25 ns plateau supports convergence of the global complex geometry within the accessible simulation time. Most protein residues showed RMSF values below 0.3 nm, consistent with overall rigidity. Peaks near residues ~100, 300, 500, and 800 reached 0.9–1.0 nm, identifying flexible loops or terminal segments. Fluctuations in the binding core were small, indicating that TVDVS binding did not disrupt protein stability. The radius of gyration (Rg) increased from ~2.90 to ~3.02 nm, with slightly larger fluctuations at 25–50 ns, and then stabilized. The absolute Rg change was limited to ~0.12 nm, which is more compatible with mild breathing-like expansion than with major unfolding or loss of compactness. Hydrogen-bond counts were high initially (11–12), then decreased gradually and stabilized at ~4–8 after 50 ns. Thus, early tight contact formation was followed by a reproducible reduction to a lower but still persistent contact state, indicating that stability was maintained by a reorganized, not vanished, interaction network. The solvent-accessible surface area (SASA) rose from ~390 to ~430 nm^2^ and then fluctuated slightly at a high level. Because the SASA increase occurred together with stable RMSD and bounded Rg values, it is better interpreted as local pocket adaptation and increased interface hydration than as destabilization of the whole complex. Overall, TVDVS exhibited stable conformation and good binding adaptability.

As shown in Figure 3c, the RMSD of ATTSI rose rapidly to ~0.2 nm within the first 20 ns and remained between 0.15 and 0.25 nm around 50 ns, indicating rapid stabilization in the binding pocket. After ~85 ns it increased toward ~0.3 nm, suggesting late-stage conformational drift or a slight adjustment of the binding mode. Importantly, even in the late stage the ligand RMSD remained below ~0.3 nm, which indicates retention of the bound state with moderate pose relaxation rather than unbinding. The complex RMSD increased to ~0.45 nm during the first 20 ns, then rose slowly to a plateau and fluctuated within 0.45–0.50 nm, indicating a relatively stable state after early adaptation. The coexistence of bounded ligand RMSD and a plateauing complex RMSD supports practical convergence of the ATTSI system after the initial equilibration phase. Protein RMSF values were generally low, with most regions below 0.3 nm. Peaks appeared at the C terminus (residues 700–800) and in a loop near residue 500, reaching ~0.75 nm. These flexible segments likely corresponded to surface loops or terminal regions and were distant from the binding core, so their impact on overall stability was limited. The radius of gyration (Rg) increased from ~2.91 to ~3.0 nm and reached a maximum between 20 and 50 ns, then decreased slightly and fluctuated at 2.98–3.0 nm. This rise and re-equilibration pattern indicates that the complex underwent a transient expansion followed by compaction into a stable conformational basin. Hydrogen-bond counts were high initially (up to ~14), then decreased and stabilized between 5 and 10. Because the later hydrogen-bond occupancy remained broad but persistent, ATTSI appears to maintain stability through a remodeled interfacial network rather than through one fixed early contact pattern. SASA increased from ~400 to 430–440 nm^2^ and then fluctuated at a high level, indicating greater solvent exposure. In combination with the bounded Rg behavior, this result suggests interface relaxation without loss of global fold integrity. In summary, ATTSI remained stably bound in the pocket for most of the simulation.

As shown in Figure 3d, the RMSD of ATTSL rose to ~0.10 nm within the first 10 ns and then remained within 0.08–0.12 nm. Late-stage stability increased, and no obvious drift was observed. Among the 6 peptides, this was the narrowest ligand RMSD interval, indicating the smallest deviation from the starting bound pose and therefore the strongest conformational retention in the simulated time window. The complex RMSD increased rapidly at the start and reached ~0.45 nm at ~20 ns. It then fluctuated slightly between 0.45 and 0.50 nm without an upward trend. This early transition into a stable plateau indicates relatively fast equilibration of the ATTSL complex compared with systems showing more persistent late drift. Most protein residues exhibited RMSF values of 0.1–0.3 nm. Higher peaks (>0.5 nm) appeared near residues ~200, 450, 650, and 800, which likely corresponded to loops or terminal segments. No high fluctuation occurred in the binding region, indicating that ATTSL did not induce marked structural disturbance. The radius of gyration (Rg) increased from ~2.90 to ~3.00 nm, reflecting modest relaxation or expansion. The trend stabilized after ~25 ns. The ~0.10 nm Rg increase indicates only limited rearrangement of the global protein scaffold and supports maintenance of compactness after binding. Hydrogen-bond counts were ~16 at the beginning and then fluctuated between 8 and 13 throughout the simulation, indicating a persistent network. This comparatively high and sustained hydrogen-bond occupancy provides the clearest quantitative support, among the 6 systems, for a robust interfacial anchoring mode. The solvent-accessible surface area (SASA) rose steadily from ~400 to ~430 nm^2^ and then reached a plateau. Because SASA, Rg, ligand RMSD, and complex RMSD all stabilized after the early stage, the trajectory supports a well-converged bound state rather than progressive rearrangement. In sum, the ATTSL complex showed high stability during the simulation.

As shown in Figure 3e, the RMSD of RSEQ rose rapidly to ~0.25 nm within the first 20 ns and then remained at 0.25–0.30 nm during 20–100 ns. This indicated that the ligand entered a stable state after early conformational adaptation. Although RSEQ exhibited a somewhat higher ligand RMSD than ATTSL or TVDVS, its fluctuation range after equilibration remained narrow, which suggests a stable but more mobile bound state rather than poor convergence. The complex RMSD increased to ~0.50 nm within the first 25 ns and then rose slowly to 0.55–0.58 nm, approaching a plateau, which indicated progressive stabilization after initial rearrangement. Thus, the RSEQ system appears to require a slightly longer relaxation period, but it still reaches a bounded late-stage regime rather than showing continuous structural divergence. Protein RMSF values were generally low, with most residues below 0.3 nm. Peaks near residues ~300, 500, and 750 approached ~1.0 nm and likely corresponded to loop or terminal regions not directly coupled to the binding site. Thus, RSEQ binding did not perturb the protein framework, and the interfacial region remained rigid and stable. The radius of gyration (Rg) increased steadily from ~2.92 to ~3.11 nm, indicating modest expansion or relaxation after binding, possibly due to pocket adjustment or increased surface exposure. Because this was the largest absolute Rg change among the 6 complexes, RSEQ may induce the strongest global relaxation response; however, the absence of abrupt jumps and the attainment of a late plateau argue against unfolding. Hydrogen-bond counts were ~11–13 at the start and decreased gradually, stabilizing at 5–9 in the late stage. This persistent late-stage hydrogen-bond population indicates that even though the initial contact pattern relaxed, a stable interaction network remained in place. The solvent-accessible surface area (SASA) rose from ~400 to ~430 nm^2^ during the first 25 ns and then fluctuated at that level. Together with the larger Rg shift, this pattern supports a more adaptive, less tightly packed complex than ATTSL, but not an unstable one. Overall, the RSEQ complex exhibited good global stability.

As shown in Figure 3f, the RMSD of ATSTLA relative to the initial conformation during the 100 ns simulation rose rapidly to ~0.25 nm within the first 20 ns and then stabilized, fluctuating between 0.20 and 0.30 nm. This indicated a rapid early conformational adjustment, followed by a stable binding mode without pronounced drift, demonstrating good positional stability at the site. The complex RMSD increased quickly to ~0.45 nm at ~20 ns, then rose slowly to ~0.55 nm and fluctuated around that level. The presence of an early rise followed by a stable high-level plateau indicates that ATSTLA underwent an induced-fit-like accommodation before reaching a persistent equilibrium state. Protein RMSF values were generally below 0.3 nm, indicating strong overall rigidity. Distinct peaks appeared near residues ~250, ~450, and ~750 (up to ~0.75 nm), suggesting flexible loops or terminal regions that might act as regulatory sites. These flexible regions could participate in conformational adaptation or pocket shaping during binding. The radius of gyration (Rg) increased slowly from ~2.95 to ~3.10 nm, indicating slight expansion of the complex over time. This ~0.15 nm increase was greater than that of ATTSIA, TVDVS, or ATTSL, implying a more pronounced global relaxation, but the change remained gradual and bounded. Hydrogen-bond counts decreased from ~10 to an average of 5–7 within the first 10 ns and then stabilized, indicating partial early breakage followed by formation of a stable new network that supported persistent interactions. The rapid transition from the initial contact-rich state to a lower but stable hydrogen-bond occupancy again supports re-equilibration rather than contact failure. The solvent-accessible surface area (SASA) increased gradually from ~400 to ~425 nm^2^, indicating greater surface exposure during binding, likely associated with protein conformational changes and the emergence of solvent-accessible regions that favored water-mediated interactions. Because SASA increased only moderately and in parallel with stable RMSD behavior, ATSTLA is best interpreted as forming a conformationally adaptive but still well-retained complex. These results supported effective binding of ATSTLA to the receptor protein.

Overall, the 6 complexes all showed the same general kinetic pattern; namely, rapid early relaxation followed by bounded fluctuations without dissociation over the remainder of the 100 ns trajectories. Quantitatively, ATTSL showed the narrowest ligand RMSD range and the most persistent hydrogen-bond network, whereas RSEQ and ATSTLA exhibited somewhat larger late-stage Rg increases and therefore stronger global relaxation responses. ATTSIA, TVDVS, and ATTSI occupied an intermediate regime in which the ligand pose remained stable while the receptor interface underwent modest adaptive rearrangement. Because only one trajectory was analyzed for each complex, these results should be interpreted as internally consistent comparative evidence of stability rather than as fully replicated ensemble statistics.

#### 3.4.2. MM-GBSA Binding Energy Analysis

As shown in Table 3, MM-GBSA was applied to MD trajectories to calculate binding energies, thereby providing an approximate post-MD estimate of relative binding favorability rather than an absolute experimental measure of receptor affinity. In the present work, the calculated ΔG values should therefore be interpreted mainly as comparative indicators among the six peptide systems. In addition, because the canonical umami ligand L-glutamate is a recognized reference ligand for the T1R1/T1R3 VFT domain, the peptide-binding results were interpreted relative to this reference binding region and not as stand-alone proof of umami activity. The binding energies (kcal/mol) for the complexes T1R1–T1R3/ATSTLA, T1R1–T1R3/ATTSI, T1R1–T1R3/ATTSIA, T1R1–T1R3/ATTSL, T1R1–T1R3/RSEQ, and T1R1–T1R3/TVDVS were −68.98 ± 2.14, −77.11 ± 3.64, −55.84 ± 3.71, −66.76 ± 2.82, −58.10 ± 0.56, and −54.74 ± 3.80, respectively (Table 3). Because the entropic contribution was not included in the MM/GBSA calculations, the resulting energies are more appropriate for relative comparison among peptides than for interpretation as absolute binding free energies. Thus, the values in Table 3 were used mainly to rank binding tendency and to support peptide-to-peptide affinity comparison. Within the MM-GBSA framework, negative values indicate thermodynamically favorable association in the simulated model, whereas more negative values suggest relatively more favorable predicted binding among the tested peptides. Accordingly, the present results support the view that all six peptides can form energetically acceptable receptor-associated states under the modeled conditions, but they do not by themselves demonstrate true biological potency or direct umami efficacy.

**Figure 4:**
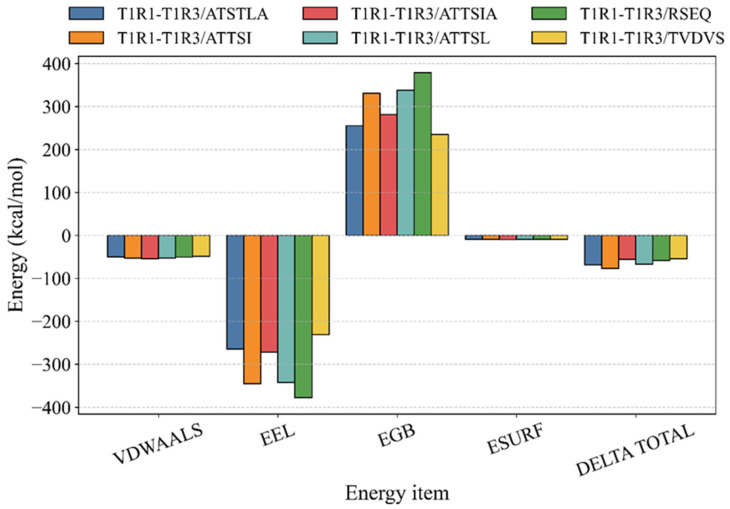
MM-GBSA binding energies and energy decomposition. Note: VDWAALS (van der Waals) denotes the molecular-mechanics van der Waals interaction energy from the force-field Lennard–Jones term; EEL (electrostatic) denotes the molecular mechanics electrostatic interaction energy (point-charge Coulomb term); EGB (polar solvation) denotes the polar solvation free energy from the generalized Born (GB) model; ESURF (nonpolar solvation / surface area) denotes the nonpolar solvation free energy; DELTA TOTAL (ΔG_bind_) represents the end-state approximation of the total binding free energy.

As shown in Figure 4, the affinity ranking of the six ligands was: ATTSI (−77.11) > ATSTLA (−68.98) > ATTSL (−66.76) > RSEQ (−58.10) > ATTSIA (−55.84) > TVDVS (−54.74) (kcal·mol^−1^). Energy decomposition indicated that the balance between electrostatics and polar desolvation determined the overall order. RSEQ showed the strongest EEL (−377.70), but it was almost completely offset by the polar solvation penalty EGB (+379.13), yielding only a moderate ΔG. ATTSI combined a pronounced EEL (−345.25) with a milder EGB (+330.81), and therefore achieved the most favorable total energy. TVDVS displayed smaller magnitudes of both EEL and EGB, which led to the least favorable total energy. Hydrophobic and steric complementarity also played a basic role. ATTSIA exhibited the most favorable VDWAALS (−54.77) and ESURF (−9.91), yet remained inferior to ATTSI, ATSTLA, and ATTSL because of electrostatic terms. These results indicate that the relative ranking among the six peptides was governed mainly by the extent to which favorable electrostatic contacts could compensate for the accompanying polar desolvation cost, while van der Waals and nonpolar solvation terms provided an important but secondary stabilizing contribution.

However, several methodological limitations of MM-GBSA should be noted. In the present calculation, configurational entropy was not explicitly included, and the resulting ΔG values therefore represent predominantly enthalpy-weighted approximations rather than full binding free energies. In addition, MM-GBSA remains sensitive to the chosen force field, implicit-solvent model, and sampled trajectory window, and is generally more reliable for relative ranking of related ligands than for absolute affinity prediction. Therefore, the numerical ΔG values reported here should not be overinterpreted as exact thermodynamic constants. Instead, they should be regarded as computational support for comparing the six candidate peptides within the same receptor model and simulation protocol. These patterns were consistent with the classic MM/GBSA view of hydrogen bonding and salt bridges, polar desolvation, and hydrophobic burial. In this context, the VFT domain of T1R1/T1R3 is more appropriately described as the predicted primary peptide-binding pocket under the present modeling framework, in qualitative agreement with the known glutamate-responsive region of the receptor, rather than as a definitively verified binding site. Ligands paired with polar residues through multipoint hydrogen bonds and salt bridges and were clamped by a peripheral hydrophobic belt. Peptides bearing acidic side chains (e.g., D/E) tended to gain stronger EEL but incurred larger EGB penalties, requiring local hydrophobic contacts and networked hydrogen bonding to balance the energy. Thus, the MM-GBSA results support a plausible and internally consistent ranking of peptide–receptor interaction favorability, but the final interpretation of umami relevance should rely on integration with the subsequent molecular dynamics, threshold, and sensory validation results rather than on ΔG values alone.

### 3.5. Analysis of Multidimensional Sensory Attributes of the Beer Body

#### 3.5.1. Multidimensional Changes over Brewing Time

All sensory data in this section were generated by the same trained panel using the unified 0–9 scoring criteria described in Supplementary Table S3, and the scores are interpreted here as mean sensory responses under a common evaluation framework [43,44,45,46,47,48]. To reduce overinterpretation of visual differences, only stage-to-stage changes that were statistically significant by one-way ANOVA are discussed as meaningful, whereas nonsignificant fluctuations are regarded as background sensory variation. On this basis, the multidimensional sensory profile of the beer changed significantly across the brewing stages (all *p* < 0.05), but the trajectories of individual attributes were not parallel. Rather, aroma, taste, and mouthfeel evolved asynchronously, indicating that the maturation of the beer body was driven by coordinated but nonidentical changes in multiple sensory dimensions.

From the initial stage to day 1 (A → B), fruity increased markedly (+2.75), whereas sweetness decreased (−2.85). Hop aroma and overall balance also declined (−1.70 and −1.80), indicating that the early fermentation stage was characterized by rapid aroma release but incomplete integration of taste and mouthfeel. During primary fermentation (B → C, 1 → 3 d), umami increased sharply (+3.10), while malty aroma, bitterness, and overall balance also rose (+1.45, +1.30, and +1.80, respectively). This pattern suggests that the sensory backbone of the beer began to emerge during active fermentation, with umami and bitterness developing in parallel rather than in opposition. During the transition from day 3 to day 10 (C → D), malty aroma continued to increase (+2.25), bitterness rose slightly (+1.25), and astringency decreased (−0.80), indicating that the beer body became fuller while the palate became cleaner. Between day 10 and day 17 (D → E), hop aroma and sweetness rebounded (+1.05 and +1.15), whereas bitterness weakened (−1.15), suggesting that the sensory profile shifted from a bitterness-supported stage toward a rounder and more integrated state. By late maturation (E → F, 17 → 29 d), sweetness and umami increased further (+3.80 and +2.15), fruity continued to rise (+1.35), and overall balance improved (+1.00), while astringency remained low at approximately 1.5. Thus, the final beer body was characterized not simply by higher intensity, but by a more coordinated profile of umami, sweetness, low astringency, and sensory harmony.

Importantly, the increase in umami during the later stages should not be attributed to a single molecular factor alone [61,65,68]. Instead, it is more appropriately interpreted as the integrated outcome of peptide evolution, residual amino acids, matrix maturation, and changing cross-modal interactions in the beer system. This interpretation is consistent with the molecular results presented above [72,75,76,77]. Section 3.1 and Section 3.2 showed that the late-stage peptide pool became relatively enriched in low- to medium-molecular-weight, less hydrophobic, and more acid/polar residue-containing peptides, whereas Section 3.3 and Section 3.4 indicated that representative candidate peptides could form stable and energetically favorable interactions with the T1R1/T1R3 model. Accordingly, the sensory rise in umami from day 10 onward was discussed here as being consistent with, but not solely proven by, the molecular evidence. The bitterness trend should also be interpreted cautiously. In this system, bitterness was not merely an adverse note but part of the structural framework of the beer body, and its transient increase during the middle stages likely contributed to palate definition before later sweetness and umami restored roundness. Likewise, the progressive decline in astringency is consistent with the known role of polyphenol–salivary protein interactions and oral lubrication in astringency perception, suggesting that matrix evolution during maturation reduced oral roughness and improved balance [78,79,80,81].

#### 3.5.2. Effects of Single Addition of Exogenous Umami Peptides on Beer Sensory Attributes

To distinguish peptide-induced sensory effects from the intrinsic sensory differences among brewing stages, all exogenous addition results were interpreted relative to the untreated control from the same stage. In other words, A-1–A-6 were compared only with A, B-1–B-6 only with B, and so forth. This stage-matched treatment was necessary because the baseline sensory profile itself changed substantially from day 0 to day 29. Accordingly, the relative changes discussed below reflect within-stage peptide effects rather than absolute differences across the full brewing timeline. Sensory responses were concentrated primarily on the umami–bitterness–aftertaste axis, whereas effects on fruity, malty aroma, hop aroma, and overall balance were generally weaker and less consistent. Relative to their corresponding same-stage controls, umami increased on average by 21.74%, bitterness by 28.41%, and aftertaste decreased by 13.33% (*p* < 0.05). By contrast, most peptide additions did not produce statistically significant effects on fruity, malty aroma, hop aroma, or overall balance (*p* > 0.05), indicating that the sensory action of these peptides was mainly taste- and mouthfeel-centered rather than aroma-driven (Supplementary Table S11).

Within individual stages, the peptide effects were directionally consistent but not identical. In the wort stage (A), ATTSIA (A-1) significantly increased umami (+0.65) and bitterness (+1.40) while shortening aftertaste (−0.70), indicating that even in a relatively immature matrix this peptide could reshape taste persistence and bitter–umami balance. RSEQ (A-5) reduced hop aroma (−0.50) and sweetness (−0.55), and ATSTLA (A-6) increased bitterness (+0.65), showing that the same peptide could express differently depending on the baseline matrix. At day 1 (B), all six peptides significantly enhanced umami (+1.15 to +1.80), with ATTSL (B-4) and ATTSI (B-3) showing the largest gains (+1.80 and +1.75). This result is notable because it indicates that the 1 d matrix was particularly responsive to exogenous peptide input. Overall balance also increased in B-4 and B-5 (+1.05 and +0.95), whereas ATTSIA (B-1) and ATSTLA (B-6) reduced fruity perception (−0.85 and −0.80), and B-6 lengthened aftertaste (+0.50). At day 3 (C), ATTSIA (C-1) and ATSTLA (C-6) significantly increased umami (+1.70 and +1.25), while several peptides reduced astringency, including C-4 (−0.80), C-5 (−0.75), and C-6 (−0.90). This suggests that at the active fermentation stage the peptides influenced not only savory perception but also oral smoothness. At day 10 (D), ATTSL (D-4) increased malty aroma and umami (+0.80 and +0.95), whereas ATSTLA (D-6) lowered hop aroma and shortened aftertaste (−0.65 and −0.55), and ATTSI (D-3) reduced bitterness (−0.70). At day 17 (E), all peptides again strengthened umami (+0.75 to +1.35), with TVDVS (E-2) and ATSTLA (E-6) showing the strongest responses. TVDVS (E-2) also reduced astringency (−0.70), ATTSIA (E-1) increased sweetness (+0.75), and ATTSI (E-3) lowered hop aroma (−0.90). At day 29 (F), when the endogenous umami background was already high, the incremental effect on umami became smaller (+0.15 to +0.45), while matrix-dependent side effects became more visible. For example, ATTSI (F-3) increased malty aroma (+0.65), TVDVS (F-2) weakened hop aroma and increased bitterness (−0.85 and +0.75), and several peptides reduced sweetness, including F-1, F-4, and F-5 (−0.70, −0.70, and −0.60). Overall balance also decreased in F-3 and F-6 (−0.95 and −0.85). Therefore, peptide addition did not act as a simple universal flavor improver. Instead, its sensory effect depended strongly on brewing stage and on the baseline composition of the beer matrix.

The mechanistic interpretation of these results should likewise be moderated and linked more explicitly to the molecular evidence. The current sensory data support a consistent role of these peptides in modulating umami, bitterness, aftertaste, and, in some cases, astringency. However, they do not support the view that all peptides act through one identical mechanism. For D/E-containing peptides such as TVDVS and RSEQ, the stronger umami-linked responses observed in some states are consistent with the molecular results showing that acidic side chains can contribute electrostatic anchoring and hydrogen-bonding interactions in the T1R1/T1R3 VFT region [75,76,77]. At the same time, the increase in bitterness seen for TVDVS in several conditions is also consistent with the broader taste-peptide literature, which links hydrophobic residues and overall hydrophobic character to bitter risk. By contrast, the S/T-rich peptides ATTSIA, ATTSI, ATTSL, and ATSTLA more likely expressed their sensory effects through hydrogen-bond-rich polar interactions and matrix-dependent modulation of mouthfeel, which may explain why some of them more often improved roundness or reduced astringency rather than producing the strongest bitter shift. Thus, the present sensory results do not indicate a simple umami-only action. Rather, they support a coupled sensory phenotype in which umami enhancement, bitterness modulation, and oral smoothing can coexist within the same peptide, with the final outcome determined by both peptide sequence and beer-matrix context.

Taken together, the sensory and molecular results converge on the same conclusion. The most relevant sequence traits in this dataset were acidic anchors, polar hydrogen-bonding capacity, and moderate hydrophobicity. These traits did not determine a single fixed sensory output, but they did shape the direction and magnitude of peptide effects on the umami–bitterness–mouthfeel axis. Therefore, the major significance of Section 3.5 is not that all six peptides behaved identically, but that they reproducibly altered the taste-centered sensory architecture of beer in a way that was broadly consistent with the receptor interaction and molecular dynamics results obtained in Section 3.3 and Section 3.4.

### 3.6. Correlation Analysis Between Umami-Peptide Cluster Distribution and Multidimensional Sensory Attributes of Lager Beer

**Figure 5:**
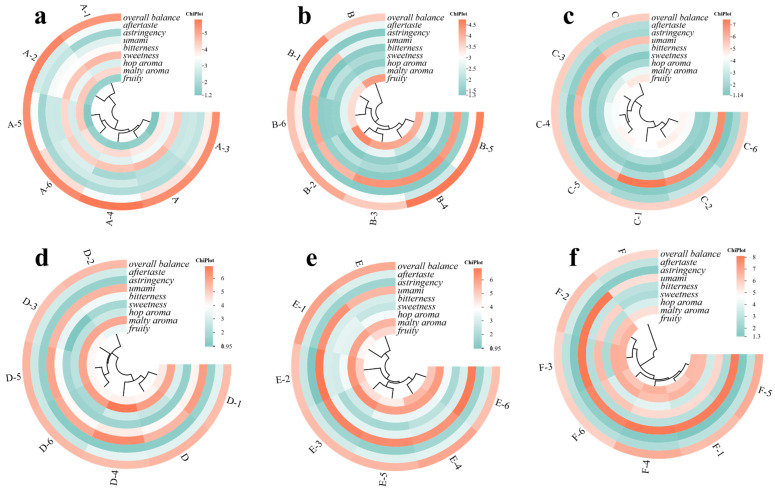

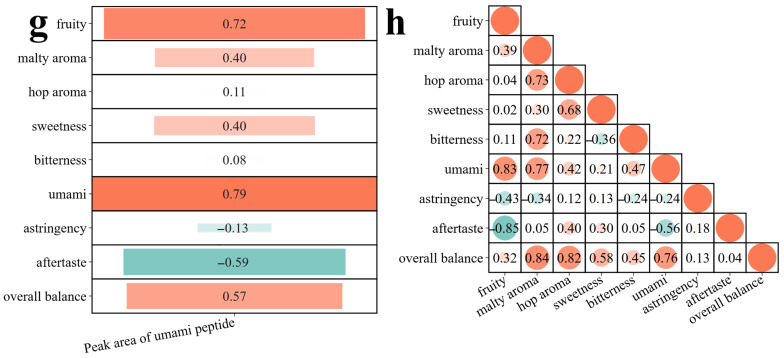
Multidimensional sensory analysis of the beer body. Multidimensional sensory evaluations for samples A–F and for samples with single additions of umami peptides (ATTSIA, TVDVS, ATTSI, ATTSL, RSEQ, ATSTLA) are labeled (**a**–**f**); (**g**) intragroup correlations among sensory dimensions; (**h**) correlations between umami-peptide peak area and the multidimensional sensory attributes of lager beer.

Using the six validated umami peptides (ATTSIA, TVDVS, ATTSI, ATTSL, RSEQ, and ATSTLA) as the core set and the remaining 80 successfully docked peptides as auxiliary candidates, only peptides that were confidently identified and exceeded the quantitative limit were retained for abundance analysis. To clarify data treatment, the stage-specific peak areas of these peptides in Supplementary Tables S12–S17 were integrated at each brewing stage to generate a stage-level abundance index, and this index was then paired with the mean sensory scores of the corresponding stage for correlation analysis. Pearson product–moment correlation analysis was used to evaluate linear associations between peptide abundance and sensory variables, and all tests were two-sided with statistical significance set at *p* < 0.05. In addition, |r| > 0.70 was interpreted as a strong correlation, 0.40 ≤ |r| < 0.70 as a moderate correlation, and |r| < 0.40 as a weak correlation. Hierarchical clustering was performed in R using Euclidean distance and the Ward.D2 agglomeration method to identify co-varying sensory modules and to visualize the relative proximity among attributes. Under this framework, the summed peak areas of the umami-peptide groups showed an initial rise, then a decline, followed by a marked increase, with time-course values of 1,221,289.25, 1,531,329.48, 2,527,617.96, 1,977,545.05, 1,226,651.74, and 3,484,753.92, respectively. This stage-dependent abundance pattern was broadly consistent with the peptide evolution results in Section 3.1, but here it was further linked quantitatively to sensory behavior. As shown in Figure 5g, the total umami-peptide peak area showed the strongest positive correlation with umami intensity (r = 0.79, *p* < 0.05), indicating that the stage-level accumulation of candidate umami peptides was most closely associated with the increase in perceived umami among all measured attributes. Positive correlations were also observed with fruity (r = 0.72) and overall balance (r = 0.57), whereas sweetness (r = 0.40) and malty aroma (r = 0.40) showed only moderate positive associations. By contrast, hop aroma (r = 0.11) and bitterness (r = 0.08) showed little direct association with peptide abundance. A clear negative correlation was observed with aftertaste (r = −0.59), while the correlation with astringency was weakly negative (r = −0.13). Importantly, these aroma-related correlations should not be interpreted as direct causal effects of peptides on aroma expression, because the single-addition experiments in Section 3.5 showed that exogenous peptide addition produced limited and inconsistent effects on fruity, malty aroma, and hop aroma. Therefore, the positive correlations of peptide abundance with fruity and overall balance are more plausibly interpreted as co-occurring process effects during fermentation and maturation, rather than as direct aroma-driving effects of the peptides themselves. In contrast, the stronger correlation with umami and the negative association with aftertaste are more consistent with the mechanistic and sensory evidence presented in earlier sections.

Based on these findings, correlations among sensory dimensions were further examined by Pearson correlation analysis and hierarchical clustering. As shown in Figure 5h, the sensory space displayed a clear fruity–umami–overall balance co-variation pattern, whereas aftertaste and astringency were positioned in the opposite direction, indicating an antagonistic sensory module. Specifically, malty aroma showed the strongest positive correlation with overall balance (r = 0.84, *p* < 0.05), and hop aroma also correlated highly with overall balance (r = 0.82, *p* < 0.05). Umami correlated positively with fruity (r = 0.83), overall balance (r = 0.76), and malty aroma (r = 0.77), supporting the existence of a positively linked sensory module centered on roundness and flavor integration. In contrast, aftertaste correlated negatively with fruity (r = −0.85), and astringency correlated negatively with fruity (r = −0.43) and malty aroma (r = −0.34), indicating that lingering constriction and residual harshness tended to oppose the rounded and integrated sensory state. Sweetness and bitterness showed a weaker antagonism (r = −0.36), suggesting that their relationship in this beer matrix was not purely binary but was modulated by the broader maturation context. The clustering result was consistent with the correlation matrix, grouping umami, fruity, malty aroma, and overall balance into one branch, while aftertaste and astringency were separated into an opposing branch. This statistical structure helps to clarify the integration between peptide abundance, sensory data, and molecular evidence. On the one hand, the positive association between peptide abundance and umami was directionally consistent with the docking and molecular dynamics results, which showed that representative short peptides could form stable interactions with the T1R1/T1R3 model. On the other hand, the near-zero correlation with bitterness indicates that peptide abundance alone did not control bitter intensity, which agrees with the single-addition experiments showing that bitterness responses were peptide-specific and matrix-dependent rather than uniformly peptide-driven. Therefore, the correlation analysis supports a more moderate interpretation. Increases in the abundance of candidate umami-peptide clusters were most directly associated with enhancement of umami and reduction in lingering aftertaste, while the apparent improvement in fruity brightness and overall balance was more likely mediated through broader fermentation-linked sensory coupling than through a direct peptide effect.

Taken together, the results suggest that umami-peptide clusters contributed most clearly to the taste-centered sensory trajectory of lager beer, especially the shift from a lingering and less integrated profile toward a rounder, more umami-prominent, and more balanced body, while the aroma-associated patterns should be interpreted cautiously as correlated maturation phenomena rather than direct molecular consequences of peptide accumulation.

## 4. Discussion and Limitation Analysis

### 4.1. Discussion

In the present study, the evidence should be interpreted at three different levels. First, machine learning screening, molecular docking, and molecular dynamics simulation are predictive tools that prioritize candidate peptides and estimate receptor compatibility trends, but they do not by themselves prove taste activity in the native beer matrix. Second, peptide occurrence in peptidomics data and the sensory changes observed after single-factor addition provide experimental support that the selected peptides are associated with changes in multidimensional sensory perception under the tested conditions. Third, what is actually demonstrated here is that a subset of screened peptides, when added individually at the tested levels, can modulate beer sensory attributes, especially umami-related and body-related perception. However, these data do not demonstrate that such peptides are the sole determinants of umami in beer, nor that in silico binding to T1R1/T1R3 is equivalent to in-mouth taste transduction in a complex beverage system. This interpretation is consistent with recent lager beer studies, which similarly combined peptidomics, computational prioritization, receptor-level simulation, and sensory verification to identify plausible umami-active peptides, while still relying on downstream validation to support, rather than absolutely prove, sensory causality [5,9,81].

Importantly, umami perception in beer should be regarded as a multicomponent phenomenon, not a peptide-only effect. Classical umami is associated with glutamate and related amino acids and is strongly potentiated by 5′-nucleotides through the T1R1/T1R3 receptor system [82]. In fermented beverages, including some beers, appreciable amounts of free glutamate can accumulate, especially under conditions of extended yeast contact [83]. At the same time, beer taste is shaped by a broader nonvolatile matrix containing amino acids, peptides, organic acids, sugars, polyphenols, and hop-derived bitter compounds [52,80,84,85]. Recent lager beer research has further shown that non-peptidic compounds, such as arginine and selected organic acids, can participate in the expression of umami-, sweetness-, bitterness-, and sourness-related taste patterns [84]. Therefore, the present results are better understood as evidence that peptides are one important contributor to beer umami and body expression, rather than the exclusive molecular basis of those perceptions [5,9,52,81,83,84].

Another issue that should be emphasized is the matrix dependence of peptide taste expression in beer. A peptide that is predicted to be umami-active in isolation may show weaker, stronger, or qualitatively shifted sensory effects once dissolved in beer, because beer already contains a strong background of bitterness, astringency, acids, ethanol-related mouthfeel, and colloidal interactions. Iso-α-acids are the major hop-derived bitter principles in beer and typically occur at concentrations on the order of 10–100 mg/L depending on beer type [85]. Polyphenols from malt and hops also contribute bitterness and astringency and participate in colloidal interactions that are closely linked to beer quality and stability [80,86]. In addition, protein/polyphenol interactions in beer are sensitive to pH and other beer constituents, which means that the dissolved, associated, or perceptually available fraction of peptides may change within the beer matrix [86,87]. For this reason, synergy and antagonism should both be considered. Classical synergy with 5′-nucleotides is mechanistically established for umami signaling [82], but in the present beer system the actual contribution of nucleotides remains unresolved because these compounds were not directly quantified. By contrast, interactions with endogenous amino acids, organic acids, polyphenols, and hop bitterness are more likely to shape the final percept. This point is also consistent with recent sensory work showing that beer body is not a single-axis property, but a multidimensional perception influenced by ethanol, viscosity, bitterness, and hop aroma [88].

The observation that some peptides classified as “umami peptides” may nevertheless increase bitterness should not be viewed as contradictory. Taste-active peptides are not always single-valence molecules; rather, their net sensory output depends on sequence composition, residue arrangement, conformation, concentration, and the surrounding matrix. Sequence-level studies have shown that umami peptides are generally enriched in acidic residues such as Asp and Glu, whereas bitterness is strongly associated with hydrophobicity and the presence or exposure of residues such as Val, Leu, Ile, Phe, and Pro [89,90,91]. Because these features can coexist in the same peptide, some molecules may display mixed umami–bitter characteristics, or their apparent taste may shift toward bitterness after addition to beer. This mechanism is particularly plausible when the umami-driving contribution of acidic residues is relatively weak, while bitterness-driving hydrophobic motifs are more dominant under beer conditions [89,90,91]. At the receptor level, previous studies have shown that some umami peptides and other umami substances can suppress bitter receptor signaling, especially hTAS2R16 [92,93]. However, this effect is not universal and depends on molecular structure and receptor subtype. Therefore, if a putative umami peptide increases bitterness in beer, the most reasonable interpretation is that its net sensory phenotype reflects the balance between its umami-related structural features, its bitterness-related hydrophobic features, and its interactions with the pre-existing beer matrix, rather than a failure of the original peptide screening logic [89,90,91,92,93].

### 4.2. Limitation Analysis

Although the present multi-evidence workflow provided coherent results from time-resolved peptidomics, computational prioritization, and sensory verification, several limitations should be acknowledged when interpreting the receptor-level conclusions. First, the docking procedure was performed on a homology-based T1R1/T1R3 model, and no experimentally resolved peptide-bound receptor structure under the same modeling framework was available for direct self-redocking or cross-docking. In structure-based studies, re-docking of cognate ligands, RMSD-based pose reproduction, and validation against reference ligands are widely used to increase confidence in docking settings and pose assignment. Therefore, the absence of explicit docking validation in the present work means that the docking results should be interpreted primarily as comparative, hypothesis-generating evidence for plausible binding modes and interaction patterns, rather than as definitive proof of the true bound conformation. This limitation does not negate the value of the receptor-level analysis, but it narrows its interpretive scope to relative structural support and candidate prioritization.

A second limitation concerns the MM/GBSA calculations. In the present study, entropy was not explicitly included because of the high computational burden and the limited robustness of routine entropy estimation in end-point free-energy workflows. However, this choice also means that the reported MM/GBSA values should not be interpreted as rigorous absolute binding free energies. Previous benchmark studies have shown that the entropic contribution can be important in protein–ligand interactions and that omission of entropy may bias the apparent favorability of binding, especially when the ligand is conformationally flexible. This point is particularly relevant for food-derived peptides, whose backbone and side-chain flexibility are substantially greater than that of many small rigid ligands. Accordingly, the MM/GBSA outputs reported here are better regarded as relative energetic descriptors within a unified computational framework, useful for ranking and comparing candidate peptides, but not for claiming exact thermodynamic binding strengths. An additional limitation of the present MD/MM/GBSA workflow is that no replicate independent MD simulations were carried out for each peptide–receptor complex. As a result, the reported dynamic behavior, MM/GBSA values, and per-residue decomposition results may still be influenced by stochastic variation associated with the initial conditions and by incomplete sampling of the accessible conformational space. This issue is especially relevant for peptide–receptor systems, in which both the ligand and the binding interface can retain considerable conformational flexibility. Moreover, although the 90–100 ns segment displayed the most stable behavior observed within the simulated time range and was therefore used for energy analysis, this time-window selection remains an operational convergence criterion within a single trajectory and does not provide the same level of methodological rigor as reproducibility demonstrated across independent runs. Future studies should therefore incorporate replicate simulations with different initial velocities, and where computationally feasible, longer or ensemble-based sampling schemes, in order to test the robustness of the trajectory-derived descriptors and the reproducibility of the MM/GBSA ranking.

Third, docking and molecular dynamics simulation do not by themselves demonstrate receptor activation or downstream signal transduction. In the present work, the computational results support the possibility that certain peptides can adopt favorable receptor contact patterns, and the sensory addition experiments further support that these peptides can modulate perception under the tested conditions. Nevertheless, receptor binding, conformational stabilization, and functional activation are not identical concepts. Current reviews on umami peptides and taste transduction have emphasized that the conformational mechanisms of T1R1/T1R3 activation are still not fully resolved, and that orthogonal methods such as heterologous cell-based receptor assays, tastebud biosensors, or advanced structural approaches are still needed for direct functional verification. Therefore, the present study demonstrates candidate relevance and sensory association, but not full receptor activation in a strict pharmacological sense.

A fourth limitation lies in the analytical and sensory design. The peptidomics data in this work are semi-quantitative rather than absolute. Although internal standard normalization improved comparability among samples, LC–MS-based peptide measurements remain susceptible to matrix-dependent ion suppression or enhancement, differential recovery, and sequence-dependent ionization efficiency. Thus, the abundance changes reported here should be interpreted mainly as relative temporal trends rather than exact endogenous peptide concentrations. In parallel, the single-addition sensory strategy was effective for isolating the directional sensory influence of selected peptides, but it cannot fully reconstruct the endogenous interaction history of peptides in naturally fermented beer. Beer taste is a matrix-dependent phenomenon, and the final sensory outcome is shaped not only by peptides, but also by amino acids, organic acids, polyphenols, iso-α-acids, and other bitter-active constituents that may interact through synergy, masking, or antagonism. For this reason, the current add-back results should be interpreted as evidence that selected peptides can influence beer perception under controlled conditions, rather than as proof that these peptides alone determine umami or bitterness expression in the native beer matrix.

Finally, the present conclusions were derived from one lager system and one production context. Beer bitterness, mouthfeel, and nonvolatile taste expression are strongly influenced by raw materials, hop regime, yeast metabolism, and process conditions, and recent lager studies have likewise shown that dominant taste attributes can shift with changes in nonvolatile acid and amino-acid composition. Therefore, while the integrated workflow proposed here is transferable in concept, the specific peptide set, their relative importance, and their sensory balance with bitterness or body may not transfer directly to other lager styles, breweries, or brewing conditions. Future work should thus extend this strategy to multiple lager matrices and independent production contexts, and combine targeted peptide quantification with receptor functional assays to further test the robustness and generalizability of the candidate peptides identified in the present study.

## 5. Conclusions

This study integrated peptidomics, receptor recognition, and quantitative sensory analysis to examine the potential links among these dimensions. RPLC-Q-TOF-MS tracked samples taken on fermentation days 0 (wort), 1, 3, 10, 17, and 29, identifying 1741, 2395, 1904, 2079, 3716, and 3515 peptides, respectively. At the algorithm and structural levels, dual thresholds (UMPred-FRL and ProUmami > 0.9) identified 112 candidate peptides; 86 were successfully docked to T1R1/T1R3. Among them, six representative peptides were selected for further validation. Their taste thresholds, docking/MM-GBSA results, and molecular dynamics behaviors collectively suggested that short peptides enriched in acidic and neutral polar residues may have relatively favorable receptor recognition potential. More importantly, sensory validation further supported the view that these peptides were not only computationally plausible candidates but also may contribute to taste perception in beer under the tested conditions. After single-peptide addition, umami increased by 21.74%, bitterness increased by 28.41%, and aftertaste decreased by 13.33% (*p* < 0.05), whereas aroma-related attributes changed little. In parallel, the summed peak area of umami-peptide groups was positively correlated with umami intensity (r = 0.79), supporting an association between peptide evolution during fermentation and multidimensional sensory expression. The main contribution of this work lies in integrating molecular occurrence, receptor-level interaction, and sensory readouts within one analytical framework for fermentation-derived peptides in lager beer. The novelty of the study is not simply the identification of candidate peptides, but the combination of computational prediction and sensory validation to support a molecule–receptor–perception relationship within a single experimental system. Overall, the results suggest that hydrophilic low- to medium-molecular-weight peptides may act as potential contributors to the final rounded and umami-prominent beer profile, and may provide a preliminary scientific basis for flavor-oriented process optimization and precise quality control of lager beer.

## Figures and Tables

**Table 1:** List of reagents and instruments.

Name/Specification	Purity/Model	Manufacturer
Acetonitrile (CAN), LC-MS grade	≥99.80% (LC-MS)	Thermo Scientific, Waltham, MA, USA.
Formic acid (FA), LC-MS grade (LiChropur, for LC-MS)	≥98–100%	Beijing Innokai Technology Co, Ltd., Beijing, China.
Ultrapure water (18.2 MΩ·cm), prepared by Milli-Q system	—	Merck Millipore, Darmstadt, Germany.
Umami peptides ATTSIA, TVDVS, ATTSI, ATTSL, RSEQ, ATSTLA, PVPL	Purity ≥ 90% (custom)	Nanjing Taopu Biotechnology Co., Ltd., Nanjing, China.
Ultrapure water system, Milli-Q	IQ 7000 series	Merck Millipore, Darmstadt, Germany.
Single-channel adjustable pipette, 100–1000 μL	—	Sinopharm Chemical Reagent Co, Ltd., Beijing, China.
Volumetric flask, 10 mL, Class A	—	Sinopharm Chemical Reagent Co, Ltd., Beijing, China.
Volumetric flask, 100 mL, Class A	—	Sinopharm Chemical Reagent Co, Ltd., Beijing, China.
Vial, 2 mL (autosampler vial)	—	Beijing Innokai Technology Co, Ltd., Beijing, China.
Vortex mixer	VM-500S	Quanan Scientific Instruments [Zhejiang] Co., Ltd., Ningbo, China.
Circulating-water vacuum pump	SHB-III	Zhengzhou Greatwall Science & Trade Co., Ltd., Zhengzhou, China.
Benchtop refrigerated microcentrifuge	Fresco™ 17	Thermo Scientific Heraeus, Waltham, MA, USA.
UPLC system	ACQUITY UPLC	Waters Corporation, Milford, MA, USA.
High-resolution QTOF mass spectrometer	TripleTOF^®^ 5600+	SCIEX, Framingham, MA, USA.

**Table 2:** Basic information and comparison of docking energies with the T1R1/T1R3 receptor for potential umami peptides.

Number	Peptide	Days	−10LogP	ALC (%)	Mass	*m*/*z*	RT	UMPred-FRL-Probability	ProUmami	ΔE_docking_ (kcal/mol)	ΔE_interaction_ (kcal/mol)	ΔE_binding_ (kcal/mol)
1	AQLPSMCRVEPQQCSIFAAGQY	0	50.12		2424.10	1213.05	40.98	0.91	1.00	——	——	——
2	AFTPLQ	0	26.23		675.36	676.36	21.35	0.97	1.00	−22.11	−52.54	−102.44
3	VGIT	0	25.58		388.23	389.24	9.88	0.93	1.00	−33.92	−44.34	−68.93
4	TIE	0	25.18		361.18	362.19	3.45	0.91	1.00	−50.03	−47.29	−37.37
5	EVA	0	22.60		317.16	318.20	3.23	0.94	1.00	−27.53	−35.38	−105.88
6	TISTM	0	20.95		551.26	552.27	11.68	0.96	1.00	−72.14	−71.69	−272.65
7	VGSVLPVFL	0	20.68		929.56	930.57	45.22	0.97	0.99	——	——	——
8	VDYNVA	0	20.65		679.32	680.32	14.82	0.91	1.00	——	——	——
9	ELT	0	20.62		361.18	362.19	8.39	0.91	1.00	−52.16	−41.39	−32.13
10	ENM	0	20.61		392.14	393.14	3.03	0.95	1.00	−74.65	−69.50	−176.00
11	TTY	0	20.60		383.17	384.17	5.03	0.94	1.00	−47.84	−46.72	−101.87
12	QLSESE	0	18.97		674.28	675.28	11.44	0.90	1.00	−70.96	−66.57	−61.68
13	ATTSIA	0	18.88		562.30	563.30	8.33	0.97	1.00	−84.19	−76.90	−288.38
14	EGAY	0	17.63		438.18	439.18	4.30	0.96	1.00	−61.03	−44.89	−9.66
15	LDVATD	0	17.54		632.30	633.31	11.17	0.97	1.00	−37.25	−43.86	−18.67
16	EIAAGLE	0	16.30		683.35	684.35	28.29	0.97	1.00	——	——	——
17	TVVL	1	24.70		430.28	431.29	25.70	0.96	1.00	−14.47	−35.93	−96.32
18	TDI	1	24.56		347.17	348.18	7.39	0.94	1.00	−48.97	−35.73	−11.91
19	EAV	1	22.57		317.16	318.17	3.24	0.98	1.00	−51.58	−40.18	−159.90
20	ESY	1	21.38		397.15	398.15	4.32	0.98	1.00	−53.11	−52.49	−146.45
21	KMT	1	21.16		378.19	379.20	12.58	0.91	0.94	−23.76	−30.92	−77.24
22	EGLA	1	21.05		388.20	389.20	6.54	0.97	1.00	−66.63	−56.15	−168.60
23	DVYVNA	1	20.56		679.32	680.32	14.77	0.94	1.00	——	——	——
24	VEY	1	19.43		409.18	410.19	12.79	0.93	1.00	−37.67	−51.25	−81.97
25	ELE	1	18.99		389.18	390.18	7.35	0.97	1.00	−13.63	−28.38	−10.59
26	AAEVLE	1	18.87		630.32	631.33	12.06	0.99	1.00	−55.69	−57.74	−121.84
27	IEVVD	1	18.14		573.30	574.30	13.83	0.95	1.00	−106.03	−77.93	−162.25
28	LEVVD	1	18.14		573.30	574.30	13.83	0.98	1.00	——	——	——
29	EATI	1	18.00		432.22	433.23	9.32	0.94	1.00	−26.74	−34.08	−41.98
30	VEVPGGLT	3	27.63		770.42	771.42	23.49	0.97	1.00	——	——	——
31	VEVPGGLTVA	3	25.56		940.52	941.52	34.14	0.94	1.00	——	——	——
32	TVSGF	3	22.91		509.25	510.25	14.27	0.95	1.00	−51.50	−53.93	−150.91
33	LEDI	3	21.55		488.25	489.25	15.02	0.90	1.00	−72.22	−54.72	−12.46
34	AATIQ	3	19.49		544.29	545.29	11.29	0.96	1.00	−59.35	−61.13	−133.67
35	AILQSVLG	3	19.32		799.48	800.48	32.37	0.94	1.00	——	——	——
36	ATTSLA	3	19.15		562.30	563.30	8.31	0.95	1.00	−74.19	−69.23	−162.70
37	TVDVSA	3	17.69		590.29	591.29	12.90	0.94	1.00	−70.74	−66.81	−61.85
38	RSEQ	3	16.19		518.24	519.25	9.43	0.94	1.00	−79.66	−78.37	−245.35
39	VCVTGF	10	35.49		624.29	625.30	27.61	0.95	1.00	——	——	——
40	AAQLPSMCRVEPQQCSIFAAGQY	10	34.14		2495.14	1248.62	41.04	0.91	1.00	——	——	——
41	AQLPSMCRVEPQQCSIF	10	31.95		1933.88	967.94	40.16	0.95	1.00	——	——	——
42	QCCQQ	10	30.96		717.22	718.23	8.07	0.92	1.00	−34.32	−48.98	−99.08
43	AAQLPSMCRVEPQQCSIF	10	24.76		2004.92	1003.42	40.38	0.94	1.00	——	——	——
44	SAGIVNS	10	22.92		646.33	647.33	8.48	0.96	1.00	——	——	——
45	TVLT	10	21.82		432.26	433.26	11.84	0.97	1.00	−58.97	−66.25	−227.06
46	DIVATD	10	20.04		632.30	633.31	11.08	0.97	1.00	−33.86	−52.72	−106.94
47	EIVDV	10	19.73		573.30	574.31	13.67	0.96	1.00	−79.30	−70.37	−142.09
48	TATY	10	18.70		454.21	455.21	4.99	0.96	1.00	−65.93	−59.37	−79.63
49	EAVT	10	17.47		418.21	419.21	2.90	0.93	1.00	−62.51	−55.80	−130.38
50	MAVTGF	10		96.30	624.29	625.30	27.61	0.92	1.00	−50.45	−61.78	−91.62
51	VSGV	17	37.01		360.20	361.21	8.57	0.94	1.00	−35.23	−40.88	−60.88
52	GVVT	17	36.44		374.22	375.22	6.09	0.95	1.00	−50.77	−44.98	−77.90
53	VDVV	17	33.20		430.24	431.25	12.28	0.94	1.00	−60.15	−54.52	−15.64
54	AEV	17	32.73		317.16	318.17	3.25	0.97	1.00	−55.34	−43.54	−53.90
55	SALP	17	32.14		386.22	387.22	10.70	0.92	0.98	−18.35	−37.55	−85.33
56	EAF	17	32.08		365.16	366.17	10.28	0.94	1.00	−48.15	−37.68	18.76
57	VVDL	17	31.91		444.26	445.27	17.23	0.96	0.97	−66.00	−65.89	−150.45
58	EVF	17	29.52		393.19	394.20	25.17	0.93	1.00	——	——	——
59	EGGVL	17	28.94		473.25	474.25	13.62	0.96	0.91	−77.17	−60.23	−165.04
60	TVGE	17	28.34		404.19	405.20	2.98	0.94	1.00	−55.38	−51.73	−122.48
61	TVES	17	27.72		434.20	435.21	10.25	0.90	1.00	−31.64	−24.55	25.66
62	LDIE	17	26.69		488.25	489.26	14.57	0.94	1.00	−51.55	−55.65	−5.19
63	TATSIA	17	26.15		562.30	563.30	8.26	0.96	1.00	−79.79	−69.15	−183.09
64	DNIY	17	25.66		523.23	524.24	11.00	0.92	1.00	−88.48	−75.14	−162.75
65	YST	17	24.58		369.15	370.16	2.48	0.96	1.00	−36.40	−50.95	−26.51
66	EQQQLNY	17	24.43		921.42	922.43	8.10	0.93	1.00	——	——	——
67	EESY	17	22.97		526.19	527.20	5.35	0.97	1.00	−92.37	−73.40	−91.95
68	TMPT	17	22.06		448.20	449.21	12.88	0.96	0.98	−54.14	−67.98	−218.12
69	LSVE	17	21.54		446.24	447.24	9.80	0.94	1.00	366.92	184.63	−19.66
70	VDYGG	17	18.25		509.21	510.22	5.80	0.94	1.00	−62.94	−60.31	−147.89
71	EPH	17	18.07		363.15	364.16	2.26	0.98	1.00	−19.38	−39.28	−67.04
72	TRST	17	16.79		463.24	464.25	24.53	0.93	1.00	−44.81	−58.88	−156.15
73	EPEP	17		94.00	452.19	453.20	4.35	0.94	1.00	−46.66	−71.82	−231.38
74	MTTVHSM	29	39.63		805.35	806.35	9.74	0.92	1.00	——	——	——
75	TVE	29	36.57		347.17	348.18	3.58	0.95	1.00	−19.66	−32.04	−96.20
76	ESL	29	35.65		347.17	348.18	6.85	0.91	1.00	−61.39	−51.19	−52.19
77	ESF	29	33.78		381.15	382.16	8.66	0.94	1.00	−65.54	−49.51	−100.96
78	EVG	29	32.77		303.14	304.15	2.71	0.97	1.00	——	——	——
79	AEL	29	31.89		331.17	332.18	8.24	0.92	1.00	−49.63	−37.80	−31.08
80	VEV	29	31.28		345.19	346.20	12.33	0.99	1.00	−58.62	−52.75	−92.63
81	EM	29	31.16		278.09	279.10	3.62	0.96	0.95	−33.61	−28.35	−59.11
82	EVQ	29	30.39		374.18	375.19	2.92	0.96	1.00	−66.65	−51.32	−91.39
83	NDT	29	30.03		348.13	349.14	7.31	0.98	1.00	−40.27	−35.64	−15.98
84	YTS	29	29.00		369.15	370.16	2.48	0.96	1.00	−30.99	−39.75	−113.51
85	SAGLVNS	29	28.90		646.33	647.34	8.59	0.95	1.00	——	——	——
86	ATTY	29	28.19		454.21	455.21	5.17	0.97	1.00	−53.47	−55.33	−199.30
87	VTY	29	26.62		381.19	382.20	14.47	0.92	1.00	−35.69	−42.30	−89.26
88	ATSTLA	29	26.02		562.30	563.30	8.23	0.95	1.00	−75.89	−68.74	−143.83
89	VEEV	29	25.99		474.23	475.24	9.38	0.98	1.00	−68.71	−53.63	−21.60
90	LSVP	29	25.71		414.25	415.25	17.05	0.98	0.99	−38.46	−56.57	−179.87
91	TVDVS	29	25.63		519.25	520.26	6.80	0.95	1.00	−87.94	−69.51	−123.89
92	ATTSI	29	25.28		491.26	492.26	8.69	0.95	1.00	−84.60	−81.85	−313.54
93	ATTSL	29	25.28		491.26	492.26	8.69	0.96	1.00	−84.22	−82.88	−350.32
94	EVVQ	29	24.50		473.25	474.25	9.24	0.97	1.00	−61.45	−64.83	−152.59
95	VTGV	29	24.48		374.22	375.22	13.71	0.96	1.00	−58.47	−50.86	−113.40
96	LTSTSP	29	23.35		604.31	605.31	9.21	0.93	1.00	−34.94	−48.73	−124.86
97	KESFKEL	29	22.96		879.47	880.47	26.48	0.92	1.00	——	——	——
98	GTGLVNS	29	21.85		646.33	647.34	8.59	0.96	1.00	——	——	——
99	EKC	29	21.69		378.16	379.16	10.04	0.91	1.00	−62.05	−63.66	−290.52
100	TATSLA	29	21.41	95.80	562.30	563.30	8.23	0.95	1.00	−53.80	−56.61	−89.46
101	GVTV	29	20.83		374.22	375.22	13.71	0.96	1.00	−45.76	−42.24	−56.00
102	VAVND	29	20.13		516.25	517.26	5.38	0.98	1.00	−63.72	−54.30	−51.30
103	FNVT	29	20.11		479.24	480.24	17.23	0.93	1.00	−36.04	−48.39	−67.88
104	VTSGF	29	19.80		509.25	510.26	14.10	0.93	1.00	−46.65	−58.53	−25.85
105	DTRVG	29	18.76		546.28	547.28	8.30	0.91	1.00	——	——	——
106	TATSI	29	18.15		491.26	492.26	8.69	0.94	1.00	−49.62	−48.50	−48.71
107	TATSL	29	18.15		491.26	492.26	8.69	0.95	1.00	−74.04	−69.04	−236.18
108	NVEVVA	29	17.80		629.34	630.34	13.69	0.94	1.00	——	——	——
109	AQLPSMCRVEPQQCSI	29	17.05		1786.82	596.61	32.11	0.90	1.00	——	——	——
110	AAGIE	29	16.64		459.23	460.24	5.17	0.94	0.98	——	——	——
111	AAGLE	29	16.64		459.23	460.24	5.17	0.94	1.00	−76.35	−57.67	−124.22
112	SERVG	29	16.41		546.28	547.28	8.30	0.96	1.00	——	——	——

Note: Days: denoted beer from fermentation day X; −10logP: confidence of spectrum-based identification for the peptide; Mass: molecular weight; m/z: mass-to-charge ratio; RT: retention time (min); ALC (%): average local confidence of the peptide; UMPred-FRL-Probabilitye, ProUmamie: probability of umami peptide; ΔE_docking_: docking energy; ΔE_interaction_: interaction energy; ΔE_binding_: binding energy. “——” indicated that the peptide did not dock successfully with the umami receptor.

**Table 3:** Binding free energies and energy components predicted by MM/GBSA (kcal/mol).

System	ΔE_vdW_	ΔE_elec_	ΔG_GB_	ΔG_SA_	ΔG_bind_
T1R1-T1R3/ATSTLA ^b^	−50.16 ± 1.59	−264.94 ± 4.56	255.28 ± 2.27	−9.16 ± 0.16	−68.98 ± 2.14
T1R1-T1R3/ATTSI ^a^	−53.22 ± 5.97	−345.25 ± 7.86	330.81 ± 9.47	−9.45 ± 0.11	−77.11 ± 3.64
T1R1-T1R3/ATTSIA ^e^	−54.77 ± 3.72	−272.23 ± 7.94	281.07 ± 4.91	−9.91 ± 0.17	−55.84 ± 3.71
T1R1-T1R3/ATTSL ^c^	−52.68 ± 2.57	−342.64 ± 9.52	337.61 ± 9.80	−9.05 ± 0.12	−66.76 ± 2.82
T1R1-T1R3/RSEQ ^d^	−50.68 ± 4.16	−377.70 ± 12.30	379.13 ± 9.84	−8.84 ± 0.05	−58.10 ± 0.56
T1R1-T1R3/TVDVS ^e^	−49.06 ± 4.53	−231.27 ± 4.56	234.83 ± 5.00	−9.24 ± 0.11	−54.74 ± 3.80

Note: ΔE_vdW_: van der Waals energy; ΔE_elec_: electrostatic energy; ΔG_GB_: electrostatic contribution to solvation; ΔG_SA_: non-polar contribution to solvation; ΔG_bind_: binding free energy; Different lowercase letters indicate significant differences in ΔG_bind_ values among different umami peptides (*p* < 0.05), whereas the same lowercase letter indicates no significant difference.

## Data Availability

The original contributions presented in this study are included in the article/Supplementary Materials. Further inquiries can be directed to the corresponding author.

## Supplementary Materials

The following supporting information can be downloaded at: https://www.mdpi.com/article/10.3390/foods15101694/s1, S1. Program for Gradient Elution; S2. PEAKS Software Settings for Peptide Identification; S3. Sensory dimensions, descriptive definitions, and scoring for beer; S4. Parameters for the identification and predicted umami intensity of peptides identified in lager beer at day 0 of the brewing process; S5. Parameters for the identification and predicted umami intensity of peptides identified in lager beer at day 1 of the brewing process; S6. Parameters for the identification and predicted umami intensity of peptides identified in lager beer at day 3 of the brewing process; S7. Parameters for the identification and predicted umami intensity of peptides identified in lager beer at day 10 of the brewing process; S8. Parameters for the identification and predicted umami intensity of peptides identified in lager beer at day 17 of the brewing process; S9. Parameters for the identification and predicted umami intensity of peptides identified in lager beer at day 29 of the brewing process; S10. Taste descriptions and thresholds of screened umami peptides; S11. Multidimensional sensory evaluation of lager beer; S12. Peptide sequences quantified in the day 0 fermentation sample and their peak areas; S13. Peptide sequences quantified in the day 1 fermentation sample and their peak areas; S14. Peptide sequences quantified in the day 3 fermentation sample and their peak areas; S15. Peptide sequences quantified in the day 10 fermentation sample and their peak areas; S16. Peptide sequences quantified in the day 17 fermentation sample and their peak areas; S17. Peptide sequences quantified in the day 29 fermentation sample and their peak areas.
